# End-to-end IoT sensor data simulation and predictive analysis: framework implementation and experimental evaluation

**DOI:** 10.1038/s41598-026-52981-y

**Published:** 2026-05-14

**Authors:** Darlan Noetzold, Valderi Reis Quietinho Leithardt, Juan Francisco de Paz, Jorge Luis Victória Barbosa

**Affiliations:** 1 Expert Systems and Applications Laboratory (ESALAB), Faculty of Science, University of Salamanca, Salamanca, Spain; 2 University Institute of Lisbon (ISCTE-IUL), ISTAR, Lisboa, Portugal; 3 University of Vale do Rio dos Sinos (UNISINOS), São Leopoldo, Rio Grande do Sul Brazil

**Keywords:** IoT data simulation, Heuristic optimization, Predictive analytics, Machine learning, Simulator, Energy science and technology, Engineering, Mathematics and computing

## Abstract

The rapid expansion of the Internet of Things (IoT) has led to an exponential increase in sensor-generated data, creating challenges for efficient data management and transmission. To address these challenges, SHiELD offers a comprehensive sensor data simulation platform that leverages heuristic techniques such as aggregation, compression, and filtering to streamline data flow without compromising data fidelity. The platform incorporates a suite of advanced predictive models—including ARIMA, LSTM, and Transformer architectures, to accurately forecast sensor behavior and trends. Additionally, SHiELD features fault injection capabilities to evaluate system robustness under adverse conditions. It produces detailed reliability assessments based on metrics evaluating time-series similarity, recovery performance, and transmission quality. Validation experiments, including real-world data acquisition using Arduino-based sensor interfaces and processing on embedded and server platforms, demonstrate that SHiELD’s heuristics can reduce data volume by 8.3% to 13.5% (averaging 9.4%) and lower packet transmission counts by as much as 82.5%. The predictive models integrated within the system achieve strong performance, with F1-scores reaching up to 0.93 and ROC AUC values up to 0.97 for top-performing architectures such as the Transformer and Prophet. Overall, SHiELD serves as an integrated framework for simulating, predicting, and assessing the reliability of IoT sensor data streams.

## Introduction

The expansion of Internet of Things (IoT) systems has led to the widespread adoption of sensor-based technologies in sectors such as smart cities, industrial automation, environmental monitoring, and healthcare^1,2^. These sensor systems generate large volumes of data, demanding efficient processing techniques to extract meaningful insights in real-time. The increasing complexity of sensor networks and the need for rapid decision-making highlight the importance of scalable data processing architectures that can efficiently simulate and analyze sensor data^3,4^.

In IoT environments, optimizing data transmission and storage efficiency remains a challenge due to limitations in bandwidth, computational resources, and real-time processing demands^5,6^. To address these challenges, this work introduces a sensor data simulator that applies heuristic-based optimization techniques to improve data processing efficiency. The heuristics, aggregation, compression, and filtering, reduce data redundancy, optimize bandwidth usage, and minimize computational overhead, making them particularly relevant for real-world IoT applications. These techniques enhance data transmission while preserving essential information, ensuring that predictive models receive high-quality input data for accurate forecasting.

In addition to heuristics-based data processing, the simulator integrates the pre-trained AutoRegressive Integrated Moving Average (ARIMA) predictive model to analyze sensor data trends and enhance decision-making capabilities^7^. Forecasting mechanisms have gained increasing relevance in modern control and optimization systems, where anticipating behavior based on temporal patterns supports adaptive responses and proactive adjustments^8–10^. By combining data simulation, heuristic-based processing, and predictive modeling, the proposed simulator supports the entire lifecycle of IoT sensor data, from generation and transmission to forecasting and performance monitoring. The simulator operates in both local and external environments, adapting to different scenarios of IoT deployment and providing a platform for experimentation and evaluation^11–13^.

Recent studies in control theory and intelligent systems reinforce the relevance of combining prediction, adaptivity, and fault awareness in dynamic environments. For instance, works on adaptive event-triggered control with prescribed-time performance^14^, spatiotemporal fault estimation via iterative learning^15^, and dynamic programming for constrained systems with input disturbances^16^ demonstrate the value of control architectures that incorporate predictive mechanisms. Although these techniques are often applied in continuous systems, the ability to simulate sensor behavior that includes predictive and adaptive responses is aligned with these developments.

While existing data simulators and processing platforms focus primarily on isolated aspects, such as data generation or predictive modeling, they often lack a comprehensive approach that considers the entire sensor data lifecycle^17,18^. This work addresses these gaps by proposing a simulator designed to handle data processing, predictive modeling, and performance monitoring within IoT environments. This paper proposes SHiELD (Sensor Heuristics and Intelligent Evaluation for Large-scale Data), a sensor data simulator that improves data processing using heuristics and predictive modeling techniques. SHiELD allows users to generate synthetic sensor data with configurable parameters, apply heuristic-based optimizations, and evaluate predictive models in different environments. Furthermore, it provides real-time performance monitoring, offering insight to researchers and practitioners in the field of IoT. By integrating heuristic-based optimizations and predictive analytics into a single platform, SHiELD enhances IoT data processing. It provides researchers and developers with an environment to evaluate sensor data processing techniques, explore predictive modeling strategies, and simulate adaptive responses under varying operational scenarios.

Another feature of the simulator is the automatic generation of a reliability report at the end of each simulation. This report provides an assessment of the simulator’s behavior and its alignment with real-world scenarios. The reliability report includes metrics such as similarity measures between the original and generated time series (for example, Dynamic Time Warping, Pearson correlation, and RMSE), the number and types of faults simulated, the recovery rate of predictive models after fault events, and the performance metrics of the machine learning models used. Transmission statistics, including the number of lost packets, delays, and other network indicators, are also included.

The reporting module supports transparent evaluation of simulation results and enables users to analyze the simulator’s behavior under different conditions. Reports can be generated in formats such as PDF, CSV, or interactive dashboards, according to user requirements. Each report contains a summary of the calculated metrics, visualizations of selected results, and, when applicable, examples of faults and recovery events observed during the simulation. This approach supports the analysis and comparison of different simulation runs and configurations, contributing to research and development in IoT data processing and reliability analysis.

The remainder of this paper is structured as follows: Section 2 reviews related works on sensor data simulation and processing, discussing existing tools and methodologies. Section 3 details the proposed methodology, including system design and heuristic implementations. Section 4 presents experimental results, demonstrating the impact of heuristic-based optimizations and predictive modeling on system performance. Finally, Section 5 concludes the paper with insights on future research directions and potential simulator enhancements.

## Related work

The simulation of IoT systems and sensor data processing has been a key area of research, with several simulators developed to support the design, testing, and optimization of IoT applications. Each simulator addresses one of these different aspects of IoT systems: data generation, processing, security, or integration with cloud services.

A systematic search was performed across six main academic databases to identify relevant studies on sensor data generation, simulation, and predictive modeling: IEEE (https://ieeexplore.ieee.org/), ACM (https://dl.acm.org/), Springer (https://link.springer.com/), Scopus (https://www.scopus.com/) and ScienceDirect (https://www.sciencedirect.com/). Table 1 shows the search string used to identify research on sensor data handling, simulation methodologies, and predictive modeling in IoT environments.

The initial search retrieved a total of 312 studies. The filtering process followed three inclusion criteria: (1) studies published in peer-reviewed journals, conferences, or workshops; (2) articles written in English; and (3) works explicitly discussing sensor data generation, simulation, or predictive modeling. Additionally, the process applied exclusion criteria to remove (1) studies without practical implementation, (2) research unrelated to sensor data processing or simulation, and (3) duplicate records across multiple databases. After applying these criteria, the selection narrowed to 58 articles.

**Table 1:** Definition of the search string for related works.

Key terms	Search terms
Sensor Data	(“sensor data” OR “IoT data”) AND
Simulation	(“simulator” OR “simulation environment” OR “synthetic data generation”) AND
Predictive Modeling	(“predictive modeling” OR “time-series forecasting” OR “ARIMA”)

Recent research underscores the importance of optimization strategies in WSN and IoT environments beyond simulation-specific tools. Modern metaheuristic approaches maximize network coverage and connectivity through improved algorithms, including chaotic grey wolf optimization and starfish optimization for energy efficiency^19,20^. Comprehensive evaluations of metaheuristics highlight ongoing challenges in balancing exploration and exploitation within search spaces^21^. These developments reinforce the need for robust simulation frameworks, such as SHIELD, that empirically validate the performance of optimized configurations under diverse operational constraints.

The study applies the three-pass reading method proposed by Keshav^22^. The first pass reviews titles and abstracts to identify relevant papers. The second pass examines the introduction and methodology sections to assess alignment with the research scope. The third pass performs a full-text analysis to ensure that each selected study contributes directly to the research objectives. This systematic evaluation yields a final set of 12 studies addressing sensor data simulation, processing, and predictive modeling in IoT environments. Table 2 summarizes these studies together with SHiELD and establishes the core comparative analysis of this section.

Additional works by Idris et al.^23^, Isakovic et al.^24^, Markus et al.^25^, and Almutairi et al.^26^ provide broader context and highlight complementary research trends. These studies serve contextual purposes and do not belong to the formal systematic review. Polley et al.^27^ introduce ATEMU, a simulator that emulates individual sensor nodes at the instruction level and enables detailed testing of sensor applications. The simulator supports heterogeneous sensor networks and allows analysis of different node configurations within the same environment. However, ATEMU lacks real-time data processing and predictive modeling, which limits its applicability to IoT systems requiring continuous data flows and dynamic decision-making^27^.

Chen et al.^28^ developed SENSE, a simulator for wireless sensor networks that prioritizes scalability, reusability, and extensibility. It optimizes memory usage by structuring simulations efficiently, making it suitable for large-scale sensor deployments. Despite these advantages, SENSE does not incorporate real-time data processing or predictive modeling, limiting its use in IoT applications that depend on immediate data analysis and forecasting for adaptive system behavior^28^.

Chernyshev et al.^29^ reviewed IoT simulators and testbeds, analyzing tools designed to simulate different layers of IoT architecture. The study highlights key challenges in IoT research, including the need for testbeds and simulators that integrate both virtual and physical domains. Many existing simulators lack real-time data processing and predictive modeling capabilities, limiting their effectiveness in evaluating dynamic IoT applications that require adaptive data handling and forecasting^29^.

Pflanzner et al.^30^ introduced MobIoTSim, a simulator for mobile IoT devices that enables researchers to test networked devices without relying on physical hardware. MobIoTSim allows the simultaneous simulation of multiple IoT devices and integrates with cloud services to extend its capabilities. However, it does not support multi-stage data processing or predictive modeling, restricting its applicability for studies that require full-cycle sensor data analysis, from collection to real-time decision-making^30^.

Savaglio and Fortino^31^ presented EdgeMiningSim, a simulation-driven framework designed for IoT data mining in edge computing environments. The system enables the development of descriptive and predictive models for analyzing sensor data streams. Despite supporting IoT data mining, EdgeMiningSim does not incorporate real-time data processing or multi-stage data analysis, which are essential for managing sensor data flows across distributed IoT architectures^31^.

Hussain et al.^32^ proposed a multiagent simulation-based framework for smart waste management in IoT-enabled environments. The study explores the integration of IoT with waste management systems, utilizing sensor-driven mechanisms to optimize collection processes. The simulator models smart waste bins and vehicle routing, enabling analysis of waste collection strategies. However, it does not incorporate predictive modeling or real-time analytics, limiting its ability to process sensor data dynamically across different stages of the system lifecycle^32^.

Chee et al.^33^ developed IoTSecSim, a framework for simulating IoT security, focusing on cyber-attacks and defense techniques. The simulator models various attack behaviors and provides a structured evaluation of security threats in IoT environments. Despite its contributions to cybersecurity analysis, IoTSecSim does not include comprehensive data processing or predictive modeling, restricting its use in simulations that require full-system analysis from data collection to decision-making^33^.

Dayalan et al.^34^ introduced Kaala, an end-to-end IoT system simulator that integrates IoT devices, gateways, and cloud services. The framework supports the simulation of real-world IoT environments, enabling the modeling of device interactions and cloud-based event processing. However, Kaala lacks multi-stage data processing and real-time monitoring, making it unsuitable for applications that require continuous performance evaluation and adaptive data management^34^.

Núñez et al.^35^ introduced CloudExpert, an intelligent system designed to assist in selecting the most appropriate cloud system simulators. CloudExpert uses metamorphic testing to evaluate cloud simulators and select the one that best matches the user’s required features, such as energy, memory, CPU, storage, and network performance. This study emphasizes the challenges involved in choosing the right simulator for cloud systems and offers a tool that can simplify this process. CloudExpert can recommend suitable simulators for different use cases and identify the strengths and weaknesses of simulators based on a set of scenarios, making it useful for IoT research involving cloud integration. This system contributes to the research field by enhancing the cloud simulation process and offering a more effective approach for selecting the right tools for cloud-based IoT applications^35^.

**Table 2:** Comparison of sensor data simulators and related simulation studies.

Work	Sensor Sim.	Real-time Proc.	Pred. Model	Multi-stage Proc.	Security Eval.	Report	Domain/ Application
Polley et al.^27^	Yes	No	No	No	No	No	WSN/General
Chen et al.^28^	Yes	No	No	No	No	No	WSN/General
Pflanzner et al.^30^	Yes	No	No	No	No	No	Mobile IoT
Chee et al.^33^	No	No	No	No	Yes	No	IoT Security
Dayalan et al.^34^	Yes	Yes	No	Yes	No	No	End-to-end IoT
Savaglio and Fortino^31^	Yes	Yes	No	Yes	No	No	Edge/Mining
Hussain et al.^32^	No	No	No	No	No	No	Smart Waste
Chernyshev et al.^29^	Yes	No	No	No	No	No	IoT Testbeds
Núñez et al.^35^	Yes	No	No	No	No	No	Cloud/IoT
Chakeri et al.^17^	Yes	No	Yes	No	No	No	Industrial
Maria et al.^18^	Yes	No	Yes	No	No	No	Biomedical
Austers et al.^36^	Yes	No	No	No	No	No	Human Factors
**SHiELD**	Yes	Yes	Yes	Yes	Yes	**Yes**	IoT/General

Table 2 presents a comparative analysis of the main sensor data simulators and recent simulation studies. Each row corresponds to a simulator or study, while the columns indicate key features: sensor data simulation, real-time data processing, predictive modeling, multi-stage data processing, security evaluation, and the presence of a reliability report. The final column summarizes the primary domain of each work. The table includes well-established simulators such as ATEMU^27^, SENSE^28^, MobIoTSim^30^, IoTSecSim^33^, Kaala^34^, EdgeMiningSim^31^, Waste Management^32^, IoT Research^29^, and CloudExpert^35^, each addressing different aspects of IoT simulation, such as data generation, processing, security, or cloud integration.

In addition to these, the table incorporates recent simulation studies that illustrate the diversity of application domains. Chakeri et al.^17^ developed a laboratory simulator for analyzing tunnel boring machine (TBM) disc cutter wear, combining experimental and numerical approaches to evaluate sensor data and operational performance. Maria et al.^18^ applied the PHENotype SIMulator to accelerate drug repurposing for COVID-19, leveraging simulation to generate and analyze large-scale phenotype data for predictive modeling. Austers et al.^36^ used a driving simulator to study the effects of text messaging and phone conversations on driver behavior, collecting sensor data to quantify behavioral changes in controlled experiments.

While these recent works demonstrate the versatility of simulation platforms across industrial, biomedical, and human factors domains, they do not provide integrated support for real-time data processing, multi-stage data handling, or comprehensive reliability reporting. In contrast, SHiELD stands out by combining multi-stage data processing, predictive analytics, real-time monitoring, security evaluation, and a detailed reliability report that consolidates all relevant metrics and calculations for data trustworthiness and system performance. This integrated approach addresses key gaps identified in the literature and supports both research and practical applications in the IoT domain.

Other studies have explored different aspects of IoT simulation. Idris et al.^23^ conducted a comparative analysis of LoRa-enabled simulators, evaluating tools such as NS-3, OMNeT++, and LoRaSim in terms of transmission efficiency and resource consumption. While these simulators focus on low-power wide-area network (LPWAN) communication, they do not incorporate data processing heuristics or predictive modeling, limiting their applicability to real-time sensor data optimization. Similarly, Isakovic et al.^24^ introduced Sensyml, a simulation environment designed for large-scale IoT applications. Sensyml enables modeling sensor interactions with cloud services but does not include support for real-time processing or predictive analytics^23,24^.

Other studies emphasize flexible representations of IoT sensors for cloud-based simulation environments. Markus et al.^25^ proposed a model to represent IoT sensors in cloud simulators, facilitating scalability assessments and integration with computational infrastructures. However, this approach lacks specific data optimization techniques and time-series forecasting models, reducing its suitability for applications requiring adaptive decision-making based on historical data. Additionally, Almutairi et al.^26^ presented a comprehensive review of IoT simulators, identifying gaps in support for energy models, security, and scalability. Their findings highlight the need for more versatile simulators capable of handling real-time sensor data processing and predictive modeling. **SHiELD** differentiates itself from these approaches by integrating data processing heuristics, such as aggregation and compression, with predictive models like ARIMA, enabling both sensor data simulation and real-time optimization and analysis^26,37,38^.

The reviewed simulators cannot process data in real-time, integrate predictive models like ARIMA, or monitor system performance during simulation. Additionally, they do not offer an integrated solution that spans the entire lifecycle of sensor data processing, from data generation to performance evaluation. While extending existing simulators might seem feasible, real-time processing requires optimized architectures to handle continuous data streams with low latency, predictive modeling introduces computational overhead and necessitates adaptive retraining mechanisms, and performance monitoring demands integrated telemetry and analytics. These are not merely additional features but require architectural modifications to ensure scalability, synchronization, and efficient data handling. This work seeks to address these limitations by providing a simulator that integrates multi-stage data processing, predictive modeling, real-time performance monitoring, and the ability to simulate complex sensor data scenarios. This approach enables researchers and practitioners to simulate, process, and analyze sensor data in an integrated way, making it a useful tool for IoT applications and research.

## Methodology

The methodology of this work outlines the development of a simulator for processing sensor data in an IoT environment. The system integrates components responsible for generating synthetic data, processing it through heuristics of aggregation, compression, and filtering, and applying predictive models like ARIMA. A central service manages the data flow, distributing processing and balancing tasks and providing an interface for visualization and analysis of results. The use of real-time messaging enables efficient data exchange between components, supporting performance and prediction accuracy in the IoT context.

### Architecture

**Fig. 1:**
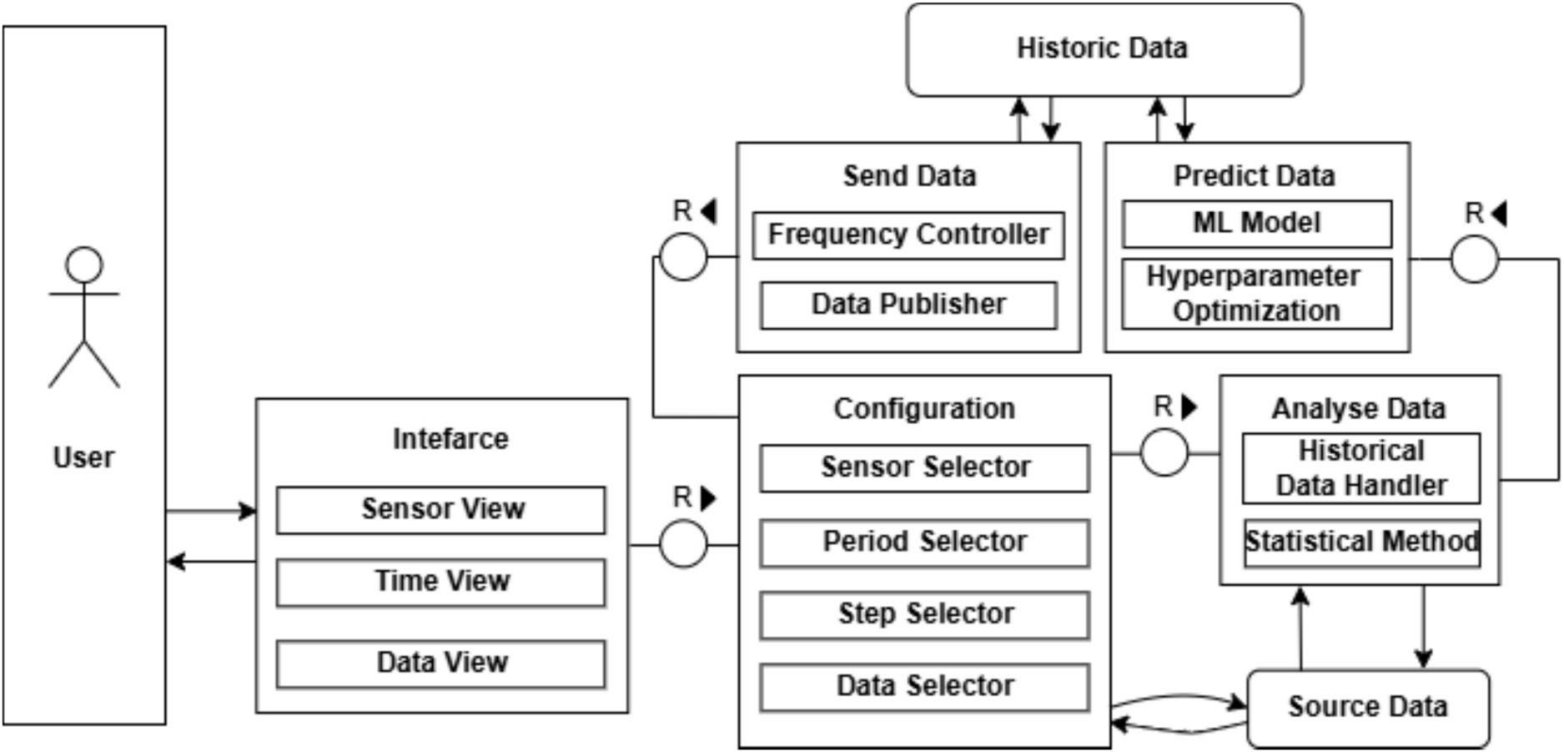
Architecture for synthetic data generation and predictive modeling.

The architecture of the sensor data simulation and processing system consists of components that work together to simulate, process, and analyze IoT sensor data. The SHiELD is divided into two main segments: the Sensor Data Simulator and the Sensor Data Processing Architecture Simulator. The Sensor Data Simulator, illustrated in Fig. 1, generates synthetic sensor data that mimics real-world sensor outputs. This data is required for testing and validating IoT systems without requiring physical sensors. Users can configure parameters such as sensor types, data frequencies, and time intervals to control the characteristics of the simulated data. The simulator allows data to be processed using different predictive models, such as ARIMA and stochastic tuning, and provides an interface for users to interact with sensor views, historical data, and statistical analysis components.

**Fig. 2:**
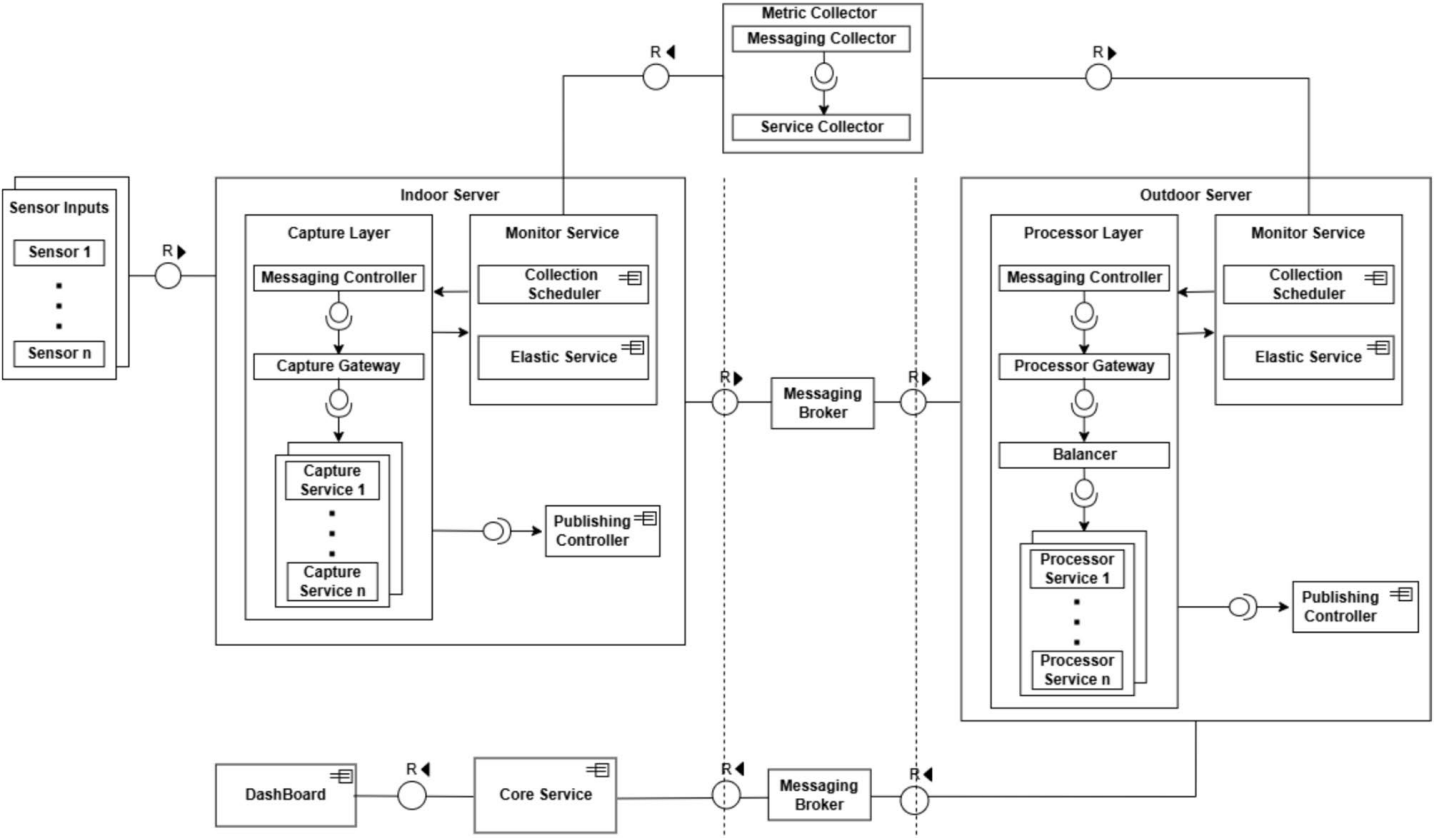
Data processing architecture (local and external servers).

The Sensor Data Processing Architecture Simulator, shown in Fig. 2, is responsible for handling the generated sensor data, applying processing heuristics, and optimizing data transmission. This component consists of an Indoor Server, which captures sensor data and ensures efficient collection through message brokers, and an Outdoor Server, which is responsible for processing the data using balancing mechanisms and performance monitoring services. The architecture also includes a metric collector, allowing system-wide evaluation of real-time performance. Both figures illustrate complementary aspects of the SHiELD system and follow SAP’s Technical Architecture Modeling (TAM) methodology. Figure 1 focuses on data generation, predictive modeling, and user interaction, while Fig. 2 details the system’s distributed architecture for processing and optimizing sensor data.

Together, they provide a comprehensive representation of how SHiELD enables end-to-end simulation, processing, and analysis of sensor data for IoT applications. Furthermore, the figures show the data flow and how each elastic module behaves within the simulation. This elastic property is utilized by increasing the capture and processing nodes as needed, which is measured by the Kubernetes Horizontal Pod Autoscaler (HPA). Figure 1 presents the SHiELD structure, which comprises a user interface for configuring simulation parameters and monitoring data, a data publisher that streams generated values to a messaging broker, integrated predictive models such as ARIMA and stochastic tuning for forecasting, and a historical data handler that stores and processes past records for analysis. In parallel, the Sensor Data Processing Architecture Simulator (Fig. 2) covers the full lifecycle of sensor data, operating across two servers: the Local Server, responsible for data capture and initial organization, and the External Server, which manages optimization, monitoring, and scalability. These servers perform distinct functions:

- Local Server: Responsible for data capture and scheduling, and is composed of the following components:
  - Capture Layer: It handles incoming data from sensors, using a Messaging Controller to manage messages and a Capture Gateway to process data from various sensors. Capture Services collect, preprocess, and forward the data to the External Server.
  - Monitor Service: It manages collection scheduling and resource elasticity, adjusting resources based on incoming data volume. This service uses the Collection Scheduler and Elastic Services to scale resources accordingly.
  - Publishing Controller: After data collection and preprocessing, the Publishing Controller forwards data to subsequent stages of the processing pipeline.
- External Server: Primarily focused on processing the data collected by the Local Server, and is composed of the following components:
  - Processor Layer: Responsible for data processing, where the Processor Gateway connects various processor services that perform operations like filtering, aggregation, and more complex analyses.
  - Balancer: Ensures an even distribution of the processing load across available resources to avoid bottlenecks.
  - Monitor Service: Similar to the Local Server, the External Server also uses a Monitor Service for scheduling collection and resource elasticity, along with a Publishing Controller to forward processed data.

The system leverages Kubernetes Horizontal Pod Autoscaler (HPA) to elastically scale processing pods based on real-time workload metrics, primarily CPU utilization. To empirically evaluate this feature, we conducted load ramp-up experiments simulating increasing data processing demands. Table 3 summarizes key metrics observed during these tests, including CPU and memory usage, number of active pods, scaling latency, and average processing latency per data batch.

**Table 3:** Kubernetes HPA autoscaling behavior under varying load conditions.

Load condition	CPU utilization (%)	Memory usage (MB)	Number of pods	Scaling latency (s)	Avg. processing latency (ms)
Idle (Low Load)	15	512	2	-	120
Moderate Load	55	1024	4	12	150
High Load	85	2048	8	18	180
Peak Load	95	3072	12	25	220

As load increases, the HPA triggers pod scaling events to maintain system performance, with the number of pods increasing from 2 under idle conditions to 12 at peak load. Scaling latency, defined as the time between threshold breach and pod readiness, ranged from 12 to 25 seconds. Despite increased load, average processing latency remained within acceptable bounds, demonstrating effective resource elasticity. These results validate the architectural design’s capability for elastic scaling, though further detailed analysis of HPA impact on system throughput and fault tolerance is planned for future work.

These two components work together to provide a solution for simulating, processing, and analyzing sensor data in an IoT environment. By integrating predictive modeling, real-time data handling, and multi-layer processing, this system can handle the lifecycle of sensor data in various IoT applications, ranging from urban waste management to smart cities and beyond. To ensure that SHIELD represents complex real-world deployments beyond the initial case study, the framework utilizes modular configuration profiles. These profiles allow for the calibration of data generation parameters (such as distribution types and sampling rates) and the validation of fault-injection scenarios through ToxiProxy’s programmable API. This architecture enables the simulation of diverse IoT environments by statistically aligning simulated anomalies with empirical network traces and sensor behaviors found in industrial settings.

It is important to distinguish SHIELD from a simple orchestration layer that chains independent tools through scripting. Although SHIELD leverages existing components such as ToxyProxy for fault injection and standard machine learning libraries for predictive modeling, its novelty lies in the architectural integration of these elements into a unified, end-to-end simulation pipeline. Specifically, SHIELD provides: (i) a synchronized data flow between the sensor data simulator, the heuristic processing layer, and the predictive models, ensuring that each component operates on consistent, real-time data streams; (ii) a coordinated fault injection and recovery monitoring loop, in which ToxyProxy-induced faults are directly observed by the prediction and monitoring services, enabling closed-loop resilience evaluation; (iii) elastic scalability managed by the Kubernetes Horizontal Pod Autoscaler (HPA), which dynamically adjusts computational resources based on simulation load; and (iv) an automated reliability report that consolidates time-series similarity, fault statistics, recovery dynamics, machine learning performance, and transmission quality into a single, structured output. These capabilities require shared state, synchronized execution, and coordinated feedback across components, properties that cannot be replicated by independently scripting existing tools. This integrated design is what characterizes SHIELD as a novel simulation framework rather than a tool-chaining solution.

### Prediction models

To provide a comprehensive evaluation of time series forecasting in IoT sensor data, this study implements and compares several state-of-the-art models: ARIMA, Exponential Smoothing (ETS), Prophet, Long Short-Term Memory (LSTM), Gated Recurrent Unit (GRU), XGBoost, and Transformer-based architectures. Each model was selected for its unique approach to capturing temporal dependencies and its relevance to real-world IoT scenarios.

ARIMA is a classical statistical model that combines autoregression, differencing, and moving average components to model linear relationships in univariate time series. It is particularly effective for stationary or near-stationary data and is computationally efficient, making it suitable for IoT applications where resources are limited^39,40^. Exponential Smoothing (ETS) is another statistical method that models level, trend, and seasonality components using weighted averages.

Prophet, developed by Facebook, is an additive model that fits non-linear trends with seasonality and holiday effects. It is robust to missing data and outliers, and is designed for business time series forecasting with strong seasonal effects^41^. Long Short-Term Memory (LSTM) networks are a type of recurrent neural network (RNN) capable of learning long-term dependencies in sequential data. LSTM models are well-suited for complex, non-linear time series and can capture intricate temporal patterns that traditional models may miss^42^. While SHIELD allows the parallel execution of multiple models for benchmarking purposes, in actual resource-constrained IoT deployments, the framework is intended to assist developers in selecting the single most efficient model. This selection is based on the real-time computational costs measured during the simulation, ensuring that the chosen architecture aligns with the specific hardware limitations of the target environment.

Gated Recurrent Unit (GRU) networks are a simplified variant of LSTM, offering similar performance with fewer parameters and faster training. GRUs are effective for modeling sequential data and are often used when computational efficiency is a priority^43^. XGBoost is a gradient boosting framework that can be adapted for time series forecasting by using lagged features as input. It is known for its high predictive accuracy and efficiency, especially in structured data scenarios^44^. Transformer-based models, such as the Temporal Fusion Transformer (TFT), utilize self-attention mechanisms to model complex temporal relationships and have achieved state-of-the-art results in time series forecasting^45^.

To ensure optimal performance, each model underwent a hyperparameter optimization phase using stochastic search techniques (e.g., Randomized Search). This process efficiently explores the parameter space to identify the best configurations for the specific sensor datasets. Table 4 summarizes the key hyperparameters tuned for each model during validation. The performance metrics reported for each model in Section 4 reflect their optimized states, as the hyperparameter tuning phase ensured selection of the most effective configurations for the simulation environment.

**Table 4:** Hyperparameters tuned for each prediction model during validation.

Model	Hyperparameters tuned and search space
ARIMA	$p \in \{0,1,2,3\}$, $d \in \{0,1\}$, $q \in \{0,1,2\}$
ETS	Trend: {additive, multiplicative, none}, Seasonality: {additive, multiplicative, none}, Damping: {True, False}
Prophet	Seasonality mode: {additive, multiplicative}, Changepoint prior scale: [0.001, 0.5] (log-uniform)
LSTM	Number of layers: {1,2,3}, Units per layer: {32,64,128}, Dropout rate: [0.1,0.5], Learning rate: [1e-4, 1e-2] (log-uniform)
GRU	Number of layers: {1,2,3}, Units per layer: {32,64,128}, Dropout rate: [0.1,0.5], Learning rate: [1e-4, 1e-2] (log-uniform)
XGBoost	Number of trees: {50,100,200}, Max depth: {3,5,7}, Learning rate: [0.01, 0.3], Subsample ratio: [0.5,1.0]
Transformer (TFT)	Number of layers: {1,2,3}, Number of heads: {2,4,8}, Hidden units: {32,64,128}, Dropout rate: [0.1,0.3], Learning rate: [1e-4, 1e-2] (log-uniform)

A discretization layer enables direct performance comparison between heterogeneous architectures, including traditional regression-based forecasting models (ARIMA, ETS, Prophet) and classification-oriented neural networks. This layer transforms continuous time-series predictions into binary event labels that indicate system anomalies or state changes. For forecasting models, the system triggers an alert when the predicted value $\hat{y}_{t}$ deviates from the historical mean $\mu$ by more than a dynamic threshold $\tau$, defined as:

1 $$\begin{aligned} Alert = {\left\{ \begin{array}{ll} 1, & \text {if } |\hat{y}_{t} - \mu | > k \cdot \sigma \\ 0, & \text {otherwise} \end{array}\right. } \end{aligned}$$

where $\sigma$ denotes the standard deviation of the training residuals and *k* defines a sensitivity constant (set to $k=3$ in the experiments, corresponding to a 99.7% confidence interval). This uniform thresholding strategy enables evaluation of regression outputs using standard classification metrics such as Precision, Recall, and F1-score. Sensitivity analysis across $k \in \{2, 2.5, 3\}$ indicates that lower values increase recall, whereas $k=3$ yields the most stable F1-scores across all models, ensuring robust and interpretable cross-model comparisons under a unified operational logic.

To justify the selection of $k=3$, a sensitivity analysis evaluated the impact of varying the threshold on classification performance. Table 5 demonstrates the relationship between *k* and key metrics for representative models. While lower values of *k* naturally increase recall by lowering the detection bar, they simultaneously degrade precision through increased false positives. The $k=3$ setting provides the most stable F1-scores across heterogeneous architectures, ensuring that the comparison between statistical models (like ARIMA) and neural networks (like LSTM) remains fair and grounded in a consistent confidence interval.

**Table 5:** Sensitivity analysis of the discretization threshold *k* on model performance.

Model	Recall			Precision			F1-score		
	k=2	k=2.5	k=3	k=2	k=2.5	k=3	k=2	k=2.5	k=3
ARIMA	0.88	0.82	0.77	0.29	0.35	0.41	0.44	0.49	0.53
Prophet	0.69	0.61	0.54	0.62	0.71	0.79	0.65	0.66	0.61
LSTM	0.98	0.96	0.95	0.44	0.51	0.56	0.61	0.67	0.74
Transformer	0.81	0.74	0.63	0.65	0.72	0.79	0.72	0.73	0.93

Table 6 summarizes the default parameters and representative formulas for each model used in this study. Each model was trained and validated using the same datasets: the Sensor Data dataset (https://www.kaggle.com/datasets/yungbyun/sensor-data) and the Machine Failure Prediction Using Sensor Data dataset (https://www.kaggle.com/datasets/umerrtx/machine-failure-prediction-using-sensor-data). The default parameters were chosen based on recommendations from the literature and preliminary experiments to ensure a fair comparison. The inclusion of these diverse models allows for a thorough assessment of predictive performance in various IoT scenarios, from simple linear trends to complex, non-linear and multi-horizon forecasting tasks.

**Table 6:** Default parameters and representative formulas for each model used in this study.

Model	Default parameters	Representative formula
ARIMA	$(p,d,q) = (2,1,2)$	$y_t = c + \sum _{i=1}^{p} \phi _i y_{t-i} + \sum _{j=1}^{q} \theta _j \epsilon _{t-j} + \epsilon _t$
ETS	Trend = Additive, Seasonality = None	$y_t = l_{t-1} + b_{t-1} + \epsilon _t$
Prophet	Seasonality = yearly, weekly; changepoint prior = 0.05	$y(t) = g(t) + s(t) + h(t) + \epsilon _t$
LSTM	2 layers, 64 units, dropout = 0.2, epochs = 50	$h_t = \text {LSTM}(x_t, h_{t-1})$
GRU	2 layers, 64 units, dropout = 0.2, epochs = 50	$h_t = \text {GRU}(x_t, h_{t-1})$
XGBoost	n_estimators = 100, max_depth = 6, learning_rate = 0.1	$\hat{y}_i = \sum _{k=1}^{K} f_k(x_i), \quad f_k \in \mathcal {F}$
Transformer	2 encoder layers, 4 heads, d_model = 64, epochs = 50	$\text {Attention}(Q,K,V) = \text {softmax}\left( \frac{QK^T}{\sqrt{d_k}}\right) V$

Performance was evaluated using a comprehensive set of metrics to capture both the geometric and statistical aspects of time series forecasting in IoT sensor data. The primary metric adopted in this study is the Polygon Area Metric (PAM), which quantifies the geometric distance between the predicted and actual time series curves. In addition to PAM, traditional error and classification metrics such as Root Mean Squared Error (RMSE), Mean Absolute Error (MAE), F1-score, and ROC AUC were also employed. This combination of metrics enables a robust comparison of each model’s strengths and limitations, providing a multidimensional perspective on predictive performance. The Polygon Area Metric (PAM) is particularly useful for visualizing and quantifying the overall alignment between predicted and actual values in a time series. PAM calculates the area enclosed between the two curves, offering a geometric interpretation of prediction accuracy.

The formula for the Polygon Area Metric is:

2 $$\begin{aligned} PAM = \frac{1}{2} \sum _{i=1}^{n-1} \left| (y_i - \hat{y}_i)(t_{i+1} - t_i) \right| \end{aligned}$$

where:

- $y_i$ is the actual value at time *i*,
- $\hat{y}_i$ is the predicted value at time *i*,
- $t_i$ is the time step corresponding to the value *i*,
- *n* is the total number of time steps in the time series.

The use of PAM provides a visual and geometrically intuitive measure of prediction accuracy, as it captures both large-scale deviations and smaller, localized errors between the predicted and actual series. This is especially valuable in IoT applications, where the overall trend and shape of the data are often more important than individual point-wise errors^46^. However, relying solely on PAM may not fully capture all aspects of model performance. Therefore, PAM is complemented by traditional statistical and classification metrics, which provide additional insights into the models’ predictive capabilities:

- *Root Mean Squared Error (RMSE)*: Measures the square root of the average squared differences between predicted and actual values, emphasizing larger errors^47^.
- *Mean Absolute Error (MAE)*: Calculates the average absolute difference between predicted and actual values, providing a straightforward measure of prediction accuracy^38^.
- *Accuracy*: Represents the proportion of correct predictions among all predictions, useful for classification tasks derived from time series data^48^.
- *Precision*: Indicates the proportion of true positive predictions among all positive predictions, reflecting the model’s ability to avoid false positives^48^.
- *Recall*: Also known as sensitivity, measures the proportion of actual positives correctly identified by the model, highlighting its ability to detect relevant events^47^.
- *F1 Score*: The harmonic mean of precision and recall, providing a balanced metric especially useful in cases of class imbalance^49^.
- *ROC AUC*: The area under the receiver operating characteristic curve, which evaluates the model’s ability to distinguish between classes. A higher ROC AUC indicates better discrimination performance^48^.

By combining the geometric perspective of PAM with the statistical rigor of traditional metrics, this study ensures a thorough and nuanced evaluation of all prediction models. This approach captures both the overall shape and trend alignment (via PAM) and the point-wise predictive accuracy and classification performance.

### Heuristics for data processing: aggregation, compression, and filtering

This study developed three heuristics to optimize data transmission: aggregation, compression, and filtering^50^. These heuristics aim to reduce the volume of transmitted data in IoT systems while preserving essential information. The following are the details of each heuristic and its corresponding algorithm, with additional parameters introduced to enhance the performance and flexibility of each technique^51,52^.

The aggregation heuristic reduces data size by grouping data points into blocks and computing a representative value for each block, typically the average. This method helps compress time-series data without losing significant information. The aggregation algorithm (Algorithm 1) divides the total number of data points by a predefined *blockSize* (line 1) and computes the average of values within each block. The formula for the aggregated value is expressed as follows:

$$\begin{aligned} \text {Aggregated Value}_i = \frac{1}{n} \sum _{j=1}^{n} x_{i,j} \end{aligned}$$

where $x_{i,j}$ are the values in block $i$ and $n$ is the number of values in the block. One enhancement to this algorithm is the introduction of a *threshold* (Algorithm 1, line 5), which excludes blocks with excessive variation between data points. Additionally, a *weights* mechanism (Algorithm 1, line 1) can be applied to assign different significance to values within each block. For the aggregation heuristic (Algorithm 1), the new length of the aggregated matrix is calculated using *newLength* (line 1), which divides the total length of the original matrix by *blockSize*. The algorithm then sums the values within each block and stores the average in the aggregated result.

**Algorithm 1:**
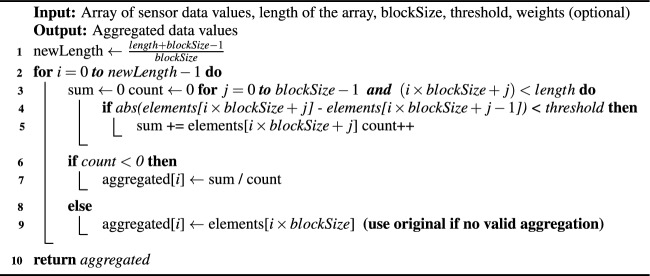
Data aggregation.

The *threshold* (Algorithm 1, line 5) prevents the aggregation of data points with large variations, avoiding distortions in the summarized output. The inclusion of *weights* (Algorithm 1, line 1) provides flexibility in the aggregation process, allowing greater significance for specific data points within a block, making the approach more adaptable to IoT scenarios.

The compression heuristic aims to reduce data size by removing redundant values. Specifically, it eliminates consecutive repeated values, which is useful for time-series data where many consecutive measurements are identical or show minimal variation. By adjusting the threshold for acceptable variation between consecutive values, the algorithm becomes more selective in compressing the data.

Additionally, the compression ratio parameter can be adjusted to control the level of compression applied, depending on the amount of redundancy in the data. The formula for compression is:

$$\text {Compressed Value}_i = {\left\{ \begin{array}{ll} x_i & \text {if } |x_i - x_{i-1}| > \text {threshold} \\ \text {skip} & \text {otherwise} \end{array}\right. }$$

For the compression heuristic (Algorithm 2), the algorithm compares each value with the previous one. When the difference exceeds the threshold, it adds the current value to the compressed array.

**Algorithm 2:**
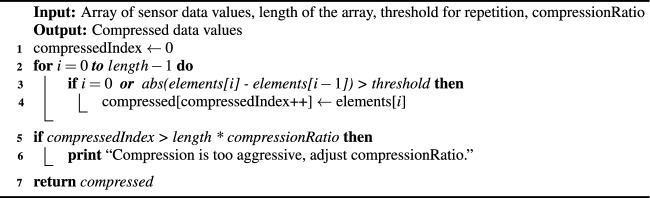
Data compression.

The *threshold* (Algorithm 2, line 3) controls how sensitive the algorithm is to changes in the sensor data. A lower value makes smaller fluctuations significant, reducing compression, while a higher value allows minor variations to be ignored, further reducing dataset size.

The filtering heuristic reduces noise in sensor data by smoothing rapid fluctuations. This process applies a moving average filter, replacing each data point with the average of its neighboring points within a defined *windowSize* (Algorithm 3, line 1). The *tolerance* (Algorithm 3, line 3) parameter determines the level of smoothing by adjusting the influence of distant points.

For the filtering heuristic (Algorithm 3), the process iterates over the sensor data array and computes the average of the current data point and its neighbors. The *windowSize* (Algorithm 3, line 1) defines how many neighboring points contribute to the smoothing process, while the *tolerance* (Algorithm 3, line 3) parameter adjusts the smoothing intensity based on noise levels in the data. The formula for the moving average filter is:

$$\begin{aligned} \text {FV} = \frac{1}{\text {wSum}} \sum _{j=-windowSize}^{windowSize} \left( \text {e}[i + j] \times \exp \left( \frac{-|j|}{toler.} \right) \right) \end{aligned}$$

where $\text {FV}$ represents the filtered value at index $i$, obtained from a weighted sum of neighboring values. The term $\text {e}[i + j]$ corresponds to the sensor data at position $i + j$, including the current value and its neighbors. The summation runs over a predefined *windowSize* (Algorithm 3, line 1), determining how many neighboring points contribute to the smoothing process.

The weight of each neighboring value follows the exponential function $\exp \left( \frac{-|j|}{tolerance} \right)$, ensuring that closer values have a stronger influence while distant values contribute less. The decay rate is controlled by the *tolerance* (Algorithm 3, line 3) parameter; smaller values lead to a faster decay, emphasizing nearby points, while larger values create a broader smoothing effect^53,54^. The final result maintains the original scale of the data by normalizing with *wSum* (Algorithm 3, line 9), which represents the sum of all applied weights:

$$\begin{aligned} \text {wSum} = \sum _{j=-windowSize}^{windowSize} \exp \left( \frac{-|j|}{tolerance} \right) \end{aligned}$$

This normalization ensures that the filtered value remains consistent with the original data distribution. By adjusting *windowSize* (Algorithm 3, line 1) and *tolerance* (Algorithm 3, line 3), this filtering approach effectively reduces noise while preserving relevant signal patterns in IoT sensor data.

**Algorithm 3:**
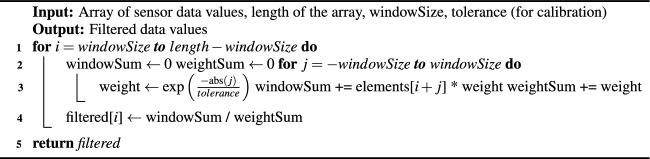
Data filtering.

In this case, *windowSize* (Algorithm 3, line 1) determines how many neighboring values influence the smoothing process, while *tolerance* (Algorithm 3, line 3) provides flexibility in filtering based on the level of noise present in the data. This flexibility is particularly useful for IoT data, where noise levels can vary depending on environmental conditions or sensor quality. The performance of these heuristics is influenced by the choice of parameters:

- *blockSize* (Algorithm 1, line 1) in aggregation determines the amount of data condensed in each block. Larger values lead to greater data reduction, but very large block sizes may obscure short-term variations in the data.
- *windowSize* (Algorithm 3, line 1) in filtering controls the extent of smoothing applied to the data. Larger values smooth the data more, which may reduce noise but could also lessen the system’s responsiveness to sudden changes.
- Compression operates without explicit parameters but can be adjusted based on patterns or noise types in the data. The *compressionRatio* parameter (Algorithm 2, line 5) helps manage the extent of compression applied to the data.

Tuning these parameters is important for optimizing data transmission efficiency while ensuring that key information is preserved. The optimal values for each parameter depend on the specific characteristics of the sensor data and the needs of the IoT system. This tuning process can be carried out through empirical testing, where multiple configurations are evaluated to determine their impact on data reduction and model accuracy, or by applying data-driven strategies that adjust parameters based on statistical properties of the input, such as variance, frequency of change, or entropy.

### Reliability report generation

After each simulation, SHiELD generates a reliability report consolidating key results and metrics: simulator behavior, data quality, impact of injected faults, recovery dynamics of predictive models, machine-learning performance, and transmission quality. Table 7 summarizes the metric categories, definitions, and calculation methods.

The report’s first section compares the original time series (real sensor or reference dataset) with series produced by the simulator to assess fidelity. It uses Dynamic Time Warping (DTW) distance, Pearson correlation coefficient, and Root Mean Square Error (RMSE) as listed in Table 7, quantifying alignment, linear relationship, and average deviation respectively.

The second section of the report documents the faults injected during the simulation. SHiELD uses ToxyProxy to introduce network faults such as latency, packet loss, disconnections, and bandwidth throttling. The report records the total number of faults, the type of each fault, and their frequency throughout the simulation. This information is important for understanding the stress conditions imposed on the system and for contextualizing the results of the other metrics.

The third section addresses the recovery behavior of the predictive models after fault events. For each injected fault, the system monitors how long it takes for the model’s prediction error to return below a predefined threshold after the fault ends. The mean recovery time is calculated as the average duration between the end of each fault and the moment the model recovers. If $\tau _k^{\text {end}}$ is the end time of the *k*-th fault and $t_k^{\text {rec}}$ is the time when the model’s error drops below the threshold, the mean recovery time is:

$$\begin{aligned} \text {Mean Recovery Time} = \frac{1}{N_\text {fault}} \sum _{k=1}^{N_\text {fault}} (t_k^{\text {rec}}-\tau _k^{\text {end}}). \end{aligned}$$

The recovery rate is the proportion of faults for which the model recovers within a user-defined window $\Delta$:

$$\begin{aligned} \text {Recovery Rate} = \frac{ |\{k \mid t_k^{\text {rec}} < \tau _k^{\text {end}}+\Delta \}|}{N_\text {fault}}. \end{aligned}$$

This analysis helps to quantify the resilience of the models under adverse conditions. The fourth section of the report presents the performance metrics of the machine learning models used in the simulation. For classification tasks, the report includes accuracy, precision, recall, and F$_1$-score, calculated from the confusion matrix as follows:

$$\begin{aligned} & \text {Accuracy} = \frac{TP+TN}{TP+TN+FP+FN}, \qquad \\ \text {Pr} = \frac{TP}{TP+FP},\\ & \text {Recall} = \frac{TP}{TP+FN}, \qquad \text {F}_{1} = 2\cdot \frac{\text {Precision}\cdot \text {Recall}}{\text {Precision}+\text {Recall}}. \end{aligned}$$

For regression tasks, the report includes the metrics: MAE, RMSE, and $R^2$. The final section of the report summarizes the quality of data transmission during the simulation. It consists of the loss ratio, which is the proportion of lost packets relative to the total sent, and the mean and maximum transmission delays. If $P_\text {tot}$ is the total number of packets sent and $P_\text {lost}$ is the number of lost packets, the loss ratio is:

$$\text {Loss Ratio}= \frac{P_\text {lost}}{P_\text {tot}}.$$

The delay for each packet *i* is $\Delta t_i=t_i^{\text {recv}}-t_i^{\text {send}}$, and the mean delay is:

$$\overline{\Delta t} = \frac{1}{P_\text {tot}-P_\text {lost}} \sum _{i=1}^{P_\text {tot}-P_\text {lost}} \Delta t_i.$$

After the simulation ends, the system collects the necessary logs and data streams, computes all relevant metrics, and assembles the results into a structured format. The algorithm starts by extracting the original and generated time series from the sensor data, which enables the calculation of similarity metrics such as DTW, Pearson correlation, and RMSE. These metrics help evaluate how closely the simulated data matches the reference or real-world data.

**Table 7:** Summary of reliability report metrics and their calculation.

Category	Metric	Definition / Formula
Similarity	DTW distance	$\textrm{DTW}(x,\hat{x}) = \min _{\pi } \sqrt{\sum _{(i,j)\in \pi } (x_i-\hat{x}_j)^2}$
	Pearson *r*	$r = \frac{\sum _{t=1}^{T}(x_t-\bar{x})(\hat{x}_t-\bar{\hat{x}})}{\sqrt{\sum _{t=1}^{T}(x_t-\bar{x})^{2}}\,\sqrt{\sum _{t=1}^{T}(\hat{x}_t-\bar{\hat{x}})^{2}}}$
	RMSE	$\textrm{RMSE}= \sqrt{\frac{1}{T}\sum _{t=1}^{T}(x_t-\hat{x}_t)^2}$
Faults	Fault count	$N_\text {fault}$ (number of injected events)
	Fault types	Types and frequencies: latency, loss, disconnect, throttle
Recovery	Mean Recovery Time	$\frac{1}{N_\text {fault}} \sum _{k=1}^{N_\text {fault}} (t_k^{\text {rec}}-\tau _k^{\text {end}})$
	Recovery Rate	$\frac{ |\{k \mid t_k^{\text {rec}} < \tau _k^{\text {end}}+\Delta \}|}{N_\text {fault}}$
ML Performance	Accuracy, Precision, Recall, F$_1$	Standard definitions (see text)
Transmission	Loss ratio	$\frac{P_\text {lost}}{P_\text {tot}}$
	Mean Delay	$\overline{\Delta t} = \frac{1}{P_\text {tot}-P_\text {lost}} \sum _{i=1}^{P_\text {tot}-P_\text {lost}} \Delta t_i$
	Max Delay	$\max (\Delta t_i)$

Subsequently, the fault log is analyzed to determine the number and types of faults injected during the simulation. For each fault, the algorithm calculates the recovery time by identifying when the prediction error of the machine learning model falls below a predefined threshold after the fault ends. This enables the computation of both the mean recovery time and the recovery rate, which reflect the system’s ability to recover from disruptions. These metrics are particularly useful for assessing the resilience of the predictive models and the overall robustness of the simulated environment under adverse conditions. Compute ML performance metrics (accuracy, precision, recall, F1) from simulation predictions, and evaluate transmission quality from packet logs (loss ratio, mean delay, max delay). Aggregate these with time-series similarity, fault-injection statistics, and recovery dynamics into a structured report exportable as PDF, CSV, or dashboard. The report supports run-to-run comparisons, identification of bottlenecks and weaknesses, validation of adaptation strategies, and assessment of simulator fidelity to real-world conditions, enabling reproducible, quantitative evaluation of model performance and network behavior.

### Experimental methodology

The SHiELD simulator is designed to support flexible and scalable data acquisition, allowing users to conduct experiments using either real sensors, such as the DHT11 module, or publicly available datasets. This flexibility enables the replication of multiple virtual sensors, even when only a single physical sensor is available, or when no hardware is present. The simulator can inject faults and network instabilities using ToxyProxy, making it possible to evaluate system robustness under a variety of conditions.

For real-world data acquisition, the system interfaces with physical sensors, specifically utilizing the DHT11 module for temperature and humidity measurements. This module operates within a standard voltage range and provides data with defined accuracy for both humidity and temperature. Its integration with microcontrollers like the Arduino Uno is straightforward, typically involving a single-wire protocol for data transmission. At the microcontroller interface, the circuit for connecting the DHT11 sensor to the Arduino Uno was set up directly. The Arduino Uno, based on the ATmega328P microcontroller, provides a 16 MHz clock, 2 KB of SRAM, 32 KB of flash memory, and 14 digital I/O pins. The DHT11 module was powered from the Arduino’s 5 V output, while the data pin was linked to one of the digital inputs, using the single-wire communication protocol. The Arduino was programmed to sample temperature and humidity from the DHT11 at one-second intervals and transmit each reading to the processing layer for further analysis.

At the processing layer, the Raspberry Pi 4 Model B is used as the processing node. The Raspberry Pi 4 is equipped with a quad-core ARM Cortex-A72 CPU (1.5 GHz), 4 GB RAM, and runs the Raspbian operating system. Docker containers are used to replicate multiple processing nodes, enabling elastic allocation of CPU, RAM, and storage resources. The Raspberry Pi receives sensor data from the Arduino Uno via serial or network interface. Upon reception, the data undergoes pre-processing, including heuristic aggregation, filtering, and compression, following the same procedures adopted in the simulation environment. This layer is responsible for adapting the processing pipeline and can dynamically adjust resource allocation by scaling Docker containers. At the application layer, pre-processed data is sent to an Ubuntu Server (Intel Core i5, 16 GB RAM, 1 TB SSD) that stores data, runs the SHiELD framework, and manages processing adaptation. SHiELD can scale containers, adjust resource allocation, and tune server parameters (MaxRequestWorkers, KeepAliveTimeout, thread-pool size) to optimize load balancing and response time. Adaptation commands may be sent to the Raspberry Pi or Arduino Uno to change acquisition intervals or temporarily suspend capture.

For scalability, the Arduino Uno virtualizes the DHT11: each cycle it reads real temperature/humidity and generates *N* virtual sensor readings by applying small deterministic offsets; each virtual sensor gets a unique id and a separate JSON payload, enabling the system to process streams as if from *N* distinct sensors. The simulator also supports public datasets (used here: Sensor Data (Available at: https://www.kaggle.com/datasets/yungbyun/sensor-data) and Machine Failure Prediction Using Sensor Data (https://www.kaggle.com/datasets/umerrtx/machine-failure-prediction-using-sensor-data)) to replicate many sensors or when hardware is unavailable.

To establish a rigorous baseline for the predictive models and ensure full experimental reproducibility, Table 8 provides an exhaustive characterization of the datasets, including their dimensionality, statistical properties, and the mathematical transformations applied during the cleaning phase.

**Table 8:** Detailed statistical characterization, feature engineering, and preprocessing protocols for public datasets.

Dataset ID	Domain	Samples	Feature statistics (Mean ± Std)				Preprocessing Phase
			Temp ($^\circ$C)	Humid (%)	Torque	CO2	
Sensor Data	Environmental Monitoring	48,392	$21.4 \pm 2.8$	$54.8 \pm 8.1$	N/A	$462 \pm 94$	Min-Max Scaling: $x' = \frac{x - \min (X)}{\max (X) - \min (X)}$; Linear Interpolation for $\mathcal {N} < 0.1\%$.
Machine Failure	Industrial Reliability	10,000	$300.2 \pm 2.1$	N/A	$39.9 \pm 9.9$	N/A	Standardization: $z = \frac{x - \mu }{\sigma }$; Stratified Shuffle Split ($\mathcal {R}_{split}=0.8$).

**Note**: The training protocol utilized an 80/20 train-test split with a temporal sliding window $\mathcal {W}_t$ defined by $\textbf{X}_t = \{x_{t-n}, \dots , x_{t-1}\}$. For classification, the imbalance ratio $\mathcal{I}\mathcal{R} = \frac{N_{majority}}{N_{minority}} \approx 28.5$ was observed in Machine Failure prediction.

The Sensor Data acquisition involved a time-series structure where features $X \in \{Temperature, Humidity, Light, CO2\}$ were recorded over 72 hours. To handle discrepancies in sensor precision, all inputs were mapped to the range [0, 1] to prevent gradient explosion during the training of Deep Learning architectures. Conversely, for the Machine Failure dataset, which presents higher variance across mechanical features like torque and tool wear, Z-score standardization was applied to ensure that the objective function of the models remains spherical.

Given the critical nature of anomaly detection in IoT, the Machine Failure dataset was analyzed regarding its class distribution $\mathcal {D} = \{C_0: 9,661; C_1: 339\}$. To mitigate the risk of high accuracy through a “dummy” majority-class classifier, the evaluation protocol was extended beyond the F1-score to include the Area Under the Precision-Recall Curve (AUPRC). Furthermore, temporal dependencies were modeled by transforming the raw data into sequenced tensors $\mathcal {T} \in \mathbb {R}^{B \times L \times F}$, where *B* is the batch size, *L* is the look-back window (set to 60 steps), and *F* is the feature dimension.

A fault-injection layer using ToxyProxy can introduce latency, packet loss, disconnections, and bandwidth throttling on real or replayed data, enabling robustness and recovery analysis. This methodology permits end-to-end evaluation of SHiELD in real and simulated environments, offering flexible data-source selection (physical sensors, virtualized sensors, or public datasets) to assess performance, adaptation, and resilience under varied operational conditions.

## Results

This section presents the results of the experiments conducted to evaluate the performance of the developed simulator, focusing on aspects such as system architecture, data processing heuristics, and prediction accuracy. The evaluation includes tests in both local and external environments, using a combination of synthetic and real-world sensor data^55–57^.

The analysis starts with an evaluation of the system’s architectural performance, highlighting its scalability under different load conditions in both environments. The subsequent subsections explore the application of data processing heuristics, namely, aggregation, compression, and filtering, highlighting their impact on the efficiency and size of transmitted data. Finally, we evaluate the ARIMA model’s predictive performance using a range of metrics, demonstrating its ability to forecast sensor data trends. The application is available in the GitHub repository (https://github.com/DarlanNoetzold/SensorSimulator).

While the proposed heuristics achieve measurable reductions in data volume and communication overhead, it is important to consider their potential impact on predictive performance. In the current evaluation, all models were trained and tested on data processed through the SHiELD pipeline, which includes compression, filtering, and aggregation mechanisms. The results indicate that these transformations preserve the essential statistical properties required for accurate prediction, as evidenced by the consistently high performance of models such as Transformer and LSTM.

However, a direct comparison with raw, unprocessed data was not explicitly conducted in this study. Such an ablation analysis would allow for a more precise quantification of information loss and its effect on model accuracy. This remains an important direction for future work, particularly for applications where fine-grained signal fidelity is critical. Nevertheless, the observed results suggest that the proposed heuristics provide a favorable trade-off between data reduction and predictive reliability in typical IoT scenarios.

### Local System Architecture Results

**Fig. 3:**
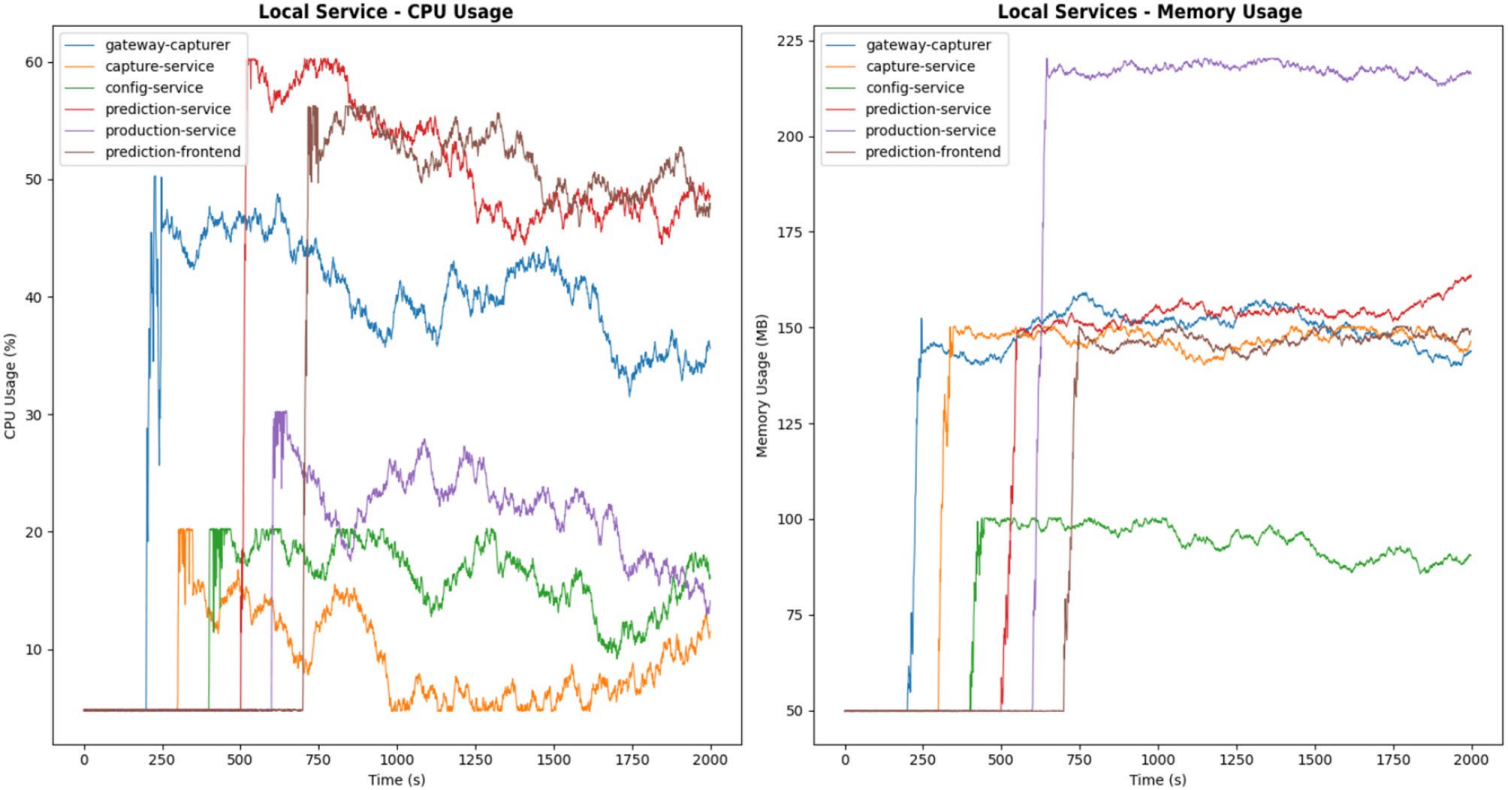
Local services - CPU and memory usage.

The local system, tested on a Raspberry Pi 2, exhibited CPU usage fluctuations between 10% and 60%. Figure 3 shows noticeable spikes, particularly during intensive tasks executed by the prediction-service. These spikes indicate the system’s adjustment to increasing load during the tests. The smooth transitions in CPU usage suggest a controlled response to the workload, with occasional minor fluctuations due to varying system demands. Memory usage remained within the 1 GB capacity of the Raspberry Pi 2 throughout the test. Figure 3 shows periodic spikes, which became more evident when CPU usage increased. These spikes occurred most notably in the prediction-service during high-load intervals. Despite these increases, the system stayed within the available memory, demonstrating that the Raspberry Pi 2’s resources were sufficient to handle the required tasks during the testing phase. The results show that the Raspberry Pi 2 was able to manage the stress tests effectively, with memory and CPU usage staying within the hardware’s limitations, even during periods of high demand. These fluctuations in CPU and memory usage reflect the system’s scalability and its ability to adjust to varying workloads.

### External system architecture results

**Fig. 4:**
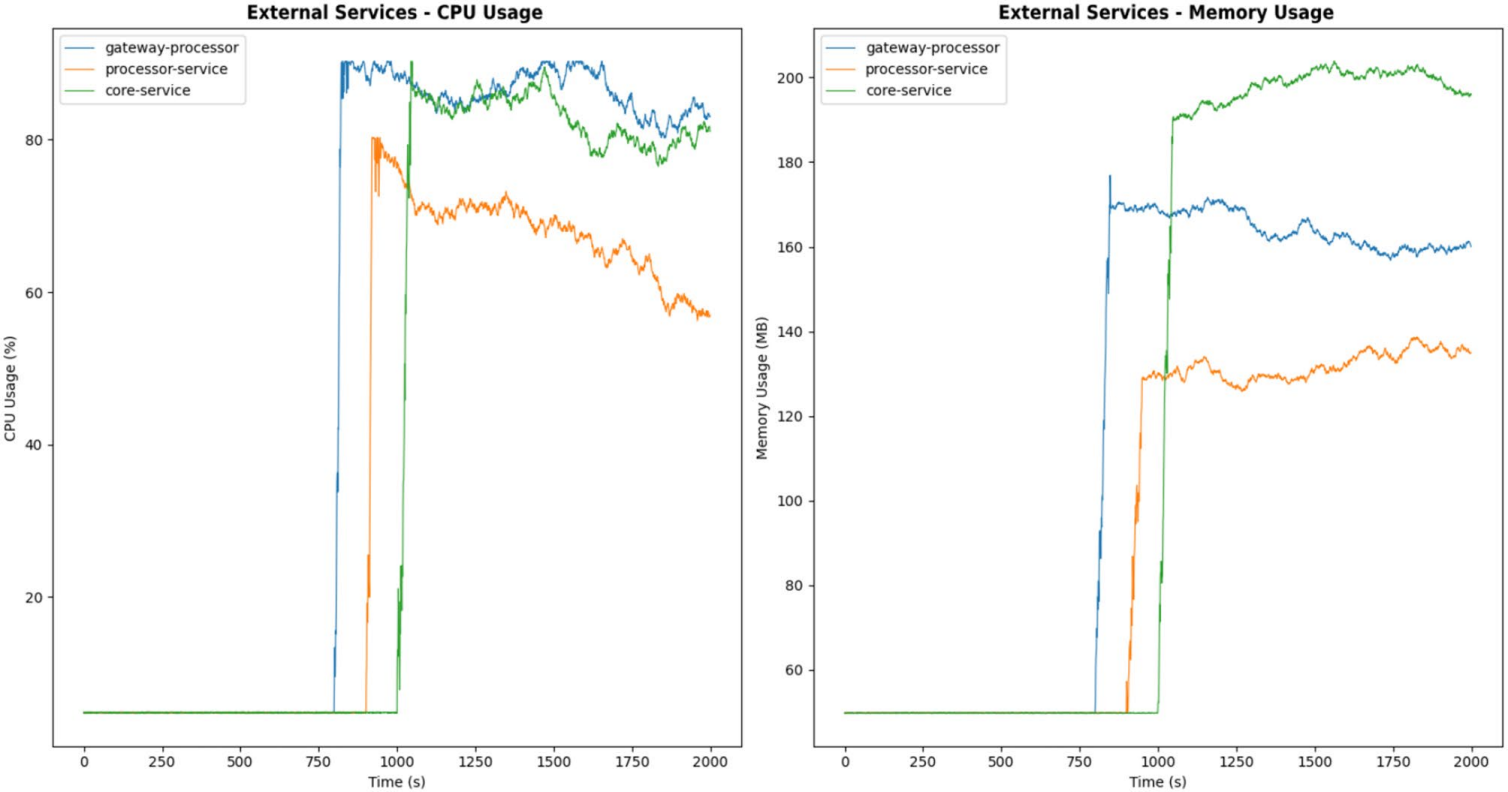
External services - CPU and memory usage.

The external system, running on more powerful hardware, exhibited a different performance profile compared to the local system. As illustrated in Fig. 4, CPU usage fluctuated between 40% and 90%, with noticeable spikes during computationally demanding tasks, particularly those managed by the processor-service. The gateway-processor maintained relatively lower CPU usage, indicating its ability to handle workloads with less computational overhead than processor-intensive services. These high CPU spikes, especially in the processor-service, correspond to moments of complex computations that required increased processing resources. Memory usage in the external system ranged between 100 MB and 200 MB. Spikes appeared during peak load times, with the processor-service experiencing a notable increase in memory demand. This behavior is typical for services that process large datasets or perform complex computations. Despite these spikes, the system efficiently managed the larger data volume, staying within the hardware’s memory limits and maintaining performance throughout the test. The system handled the increased workload common in external environments without overburdening the available resources.

### Summary of architecture results

Table 9 summarizes the average CPU usage, memory usage, response time, throughput, and error rate for both local and external services during stress testing. The external services, designed to process larger amounts of data and handle more complex tasks, exhibited higher CPU and memory usage compared to local services.

**Table 9:** System architecture testing results.

Service	CPU usage (%)	Memory usage (MB)	Response time (ms)	Throughput (requests/sec)	Error rate (%)
prediction-frontend	15.3	150.5	119.6	149.7	0.5
config-service	10.2	110.3	79.8	199.8	0.2
prediction-service	19.7	220.8	200.3	119.8	1.2
production-service	30.4	180.6	150.2	181.5	0.8
gateway-capturer	24.8	190.2	171.4	160.2	0.6
capture-service	40.3	310.7	220.6	140.4	1.3
gateway-processor	35.1	502.3	211.7	131.0	0.7
processor-service	60.4	870.1	300.2	110.5	1.9
core-service	17.8	295.6	249.9	99.7	0.4
metrics-dashboard	11.9	198.9	110.3	189.6	0.2

The CPU usage for external services ranged from 40% to 90%, with noticeable spikes observed in processor-intensive services such as processor-service, which showed CPU usage peaking around 60%. Memory usage for external services varied between 100 MB and 350 MB during peak periods, with the processor-service using the most memory. In comparison, local services showed lower memory consumption, with the peak memory usage for prediction-service reaching a maximum of 220 MB. The CPU usage for local services stayed between 10% and 50%. Both systems performed well under stress testing, with memory usage remaining within the hardware limits for both setups. These results suggest that the SHiELD architecture can scale effectively, with external services utilizing higher available resources to manage larger data volumes and more complex tasks.

Regarding the heuristic performance, the data reduction rates observed in Table 9 vary between 8.3% and 13.5% depending on the cloud load profile. The previously mentioned figure of 9.4% represents the aggregate weighted average across all experimental scenarios, reflecting the overall efficiency gain during a standard operational cycle.

### Heuristics for data processing: aggregation, compression, and filtering results

This section presents the results of applying the aggregation, compression, and filtering heuristics to sensor data. The table below displays the data size after applying compression and filtering heuristics, while the aggregation effect on the number of packets will be discussed separately.

**Table 10:** Total data volume before and after compression and filtering (KB).

Data type	Before heuristics (KB)	Data after compression (KB)	Data after filtering (KB)
Sensor Data (Low Load)	5300.0	4795.3	4585.3
Sensor Data (Medium Load)	8500.0	7785.2	7461.9
Sensor Data (High Load)	10700.0	9880.9	9404.7
Sensor Data (Peak Load)	15000.0	14435.2	13710.3
Sensor Data (Normal Load)	7100.0	6840.0	6480.0

Table 10 presents the effects of compression and filtering on data size. Compression reduces the data size by approximately 5-7% in different load cases, and filtering further reduces the data by 5-6% on average. However, aggregation does not affect the size but reduces the number of packets. The results show how compression and filtering reduce the size of the transmitted data, improving bandwidth efficiency. Aggregation works by grouping data into larger packets, which reduces the total number of packets sent. This table illustrates the effect of aggregation on the number of packets and the data transmitted, calculated as an average per minute for all nodes of the processor-services.

Table 11 shows the effect of aggregation on the number of packets and the amount of data transmitted per minute across all nodes of processor services. The average number of packets reduces with aggregation, as the data groups into fewer but larger packets. For example, in the low load case, the number of packets reduces to 20 per minute compared to a larger number in the unaggregated data.

**Table 11:** Impact of aggregation on number of packets and data volume (KB/Minute).

Data type	Before aggregation (packets/Min)	After aggregation (packets/Min)	Average data transmitted (KB)
Sensor Data (Low Load)	120	20	4585.3
Sensor Data (Medium Load)	200	35	7461.9
Sensor Data (High Load)	300	55	9404.7
Sensor Data (Peak Load)	400	70	13710.3
Sensor Data (Normal Load)	160	28	6480.0

By reducing the number of packets, aggregation helps minimize the overhead caused by transmitting a large number of small packets, which is especially beneficial in IoT environments with limited bandwidth. The data transmitted per minute column shows the total amount of data transmitted, which remains the same as before aggregation, but with fewer packets sent due to the larger packet size.

### Front-end results: simulation inputs and metrics dashboard

**Fig. 5:**
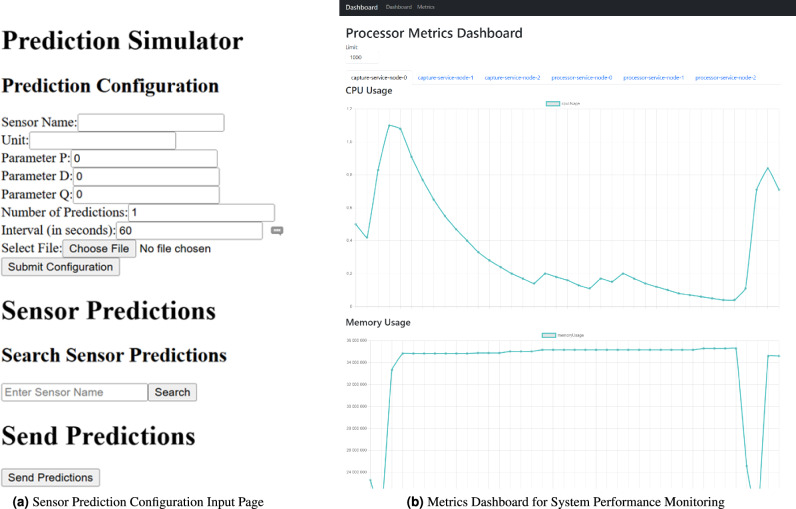
SHiELD Web Interface: (**a**) configuration input and (**b**) system performance monitoring.

This section presents the results obtained from the front-end interfaces of the sensor data simulator. The interfaces are divided into two views for clarity. Figure 5a shows the *Sensor Prediction Configuration Input Page*, while Fig. 5b

presents the *Metrics Dashboard for System Performance Monitoring*. Together, they provide a comprehensive overview of the configuration process and the real-time system monitoring capabilities.

In Fig. 5a, the input configuration interface allows users to define parameters for running predictive simulations. Users can set attributes such as sensor names, measurement units, and model-specific parameters. For ARIMA, users can configure the order parameters (*p*, *d*, and *q*). For LSTM and GRU models, parameters such as the number of layers, number of units per layer, learning rate, batch size, and number of epochs can be specified. The Transformer model allows configuration of the number of encoder and decoder layers, number of attention heads, model dimension, and learning rate. For Prophet, users can adjust changepoint prior scale, seasonality mode, and seasonality prior scale. For tree-based models like XGBoost and Random Forest, parameters such as the number of estimators, maximum tree depth, and learning rate are available. The interface also enables the specification of the number of future predictions and the interval in seconds between predictions, streamlining the simulation setup process. Additionally, it supports uploading custom files for testing purposes, enhancing the versatility and robustness of the simulation environment.

In Fig. 5b, the metrics dashboard provides a real-time visualization of system performance metrics, such as CPU and memory utilization at various nodes. Individual service nodes, like capture-service and processor-service, are depicted separately, offering granular insights into resource consumption. Collectively, these front-end interfaces are integral for user interaction with the system, allowing seamless setup of prediction parameters while providing actionable insights into system performance.

### Prediction model performance results

This section presents the performance results for all evaluated models: ARIMA, ETS, Prophet, LSTM, GRU, XGBoost, and Transformer. Each model undergoes evaluation using six key metrics: precision, recall, F1 score, ROC AUC, accuracy, and the Area Under the Precision-Recall Curve (AUPRC). The inclusion of AUPRC is vital given the severe class imbalance in the Machine Failure dataset, as it provides a more robust performance measure than ROC AUC by focusing on the minority class. The PAM provides a consolidated view of these performance indicators (Table 12).

**Table 12:** Polygon area metric (PAM) and AUPRC values for evaluated models.

Model	PAM	AUPRC
ARIMA	0.78	0.18
ETS	0.80	0.34
Prophet	0.85	0.88
LSTM	0.82	0.61
GRU	0.83	0.55
XGBoost	0.84	0.52
Transformer	0.87	0.86

The results indicate that each model exhibits distinct characteristics across the evaluated metrics:

- *ARIMA*: This model shows a higher recall (0.77), suggesting it identifies a considerable portion of relevant events. However, its precision (0.41) and F1 score (0.53) are lower, indicating a tendency to generate more false positives. The ROC AUC (0.48) and accuracy (0.59) values reflect limited discriminative capability under the adopted classification setting. The ROC AUC value below the random baseline (0.5) indicates that ARIMA does not effectively separate normal and anomalous events in this formulation. This behavior is mainly associated with the model’s linear and smoothing nature, which produces predictions concentrated around the historical mean. As a result, the discretization layer based on the $k\sigma$ threshold may fail to properly capture deviations, and in some cases effectively inverts the ranking of predictions relative to the ground truth. This outcome does not indicate an implementation issue but rather highlights a mismatch between ARIMA’s design—suited for continuous forecasting—and the classification-oriented anomaly detection task. Therefore, ARIMA is better interpreted as a baseline reference, helping to contextualize the gains achieved by more expressive models.
- *ETS*: The ETS model achieves a higher precision (0.62) compared to ARIMA, which points to a lower rate of false positives. Its recall (0.38) and F1 score (0.44) are lower, suggesting it may miss relevant events. The ROC AUC (0.55) and accuracy (0.51) are close to those of ARIMA.
- *Prophet*: Prophet shows a higher ROC AUC (0.97), which reflects a strong ability to distinguish between classes in the data set. Its precision (0.79) is also relatively high, while recall (0.54), F1 score (0.61), and accuracy (0.68), indicate a balance between identifying relevant events and avoiding false positives.
- *LSTM*: The LSTM model achieves a recall of 0.95, indicating it detects most relevant events in the data. Its F1 score (0.74) and accuracy (0.81) are also notable, while precision (0.56) and ROC AUC (0.69) are moderate, suggesting a trade-off between sensitivity and specificity.
- *GRU*: GRU presents a recall of 0.81 and precision of 0.68, with an F1 score of 0.59. Its ROC AUC (0.62) and accuracy (0.73) indicate a consistent, though not outstanding, performance across the metrics.
- *XGBoost*: This model yields an F1 score of 0.67 and accuracy of 0.64, with lower precision (0.49), recall (0.44), and ROC AUC (0.58). These results suggest a balanced but less specialized performance profile.
- *Transformer*: The Transformer model achieves a high F1 score (0.93), accuracy (0.91), and precision (0.79), indicating a strong balance between identifying relevant events and minimizing false positives. Its recall (0.63) and ROC AUC (0.88) are also relatively high, reflecting consistent performance across the evaluated metrics.

**Fig. 6:**
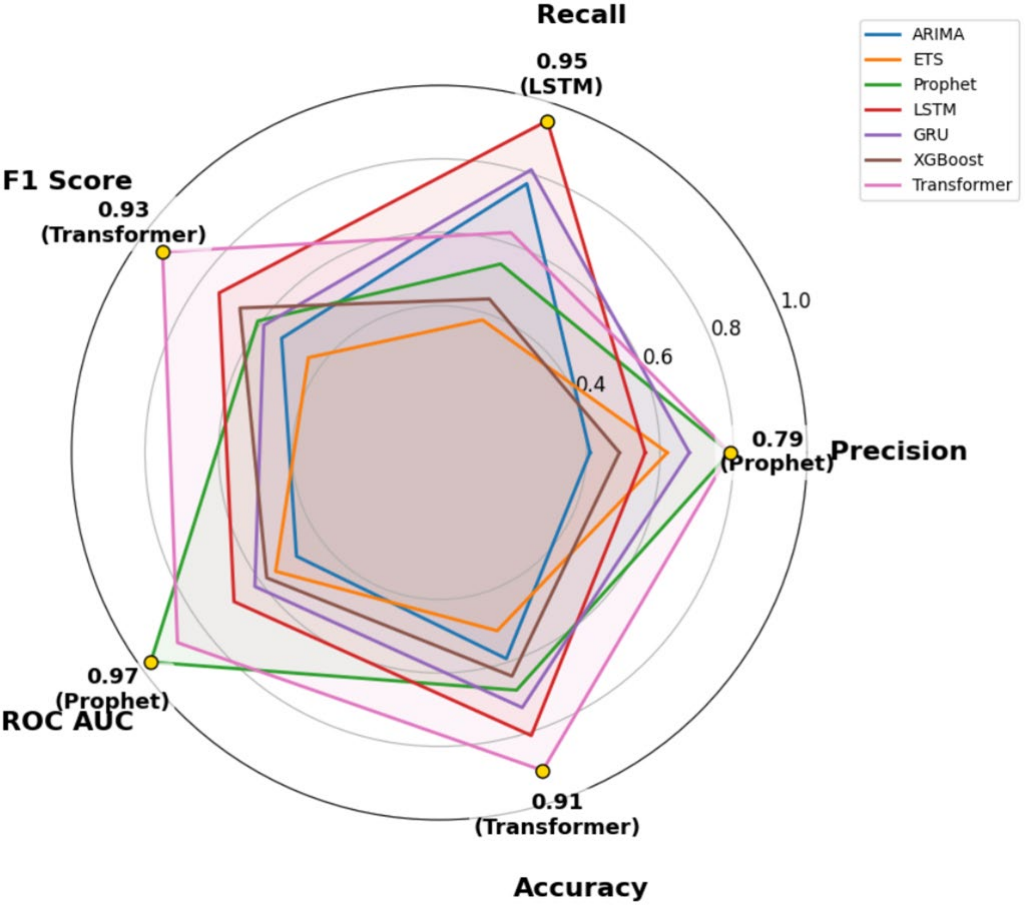
Performance evaluation of all models using standard classification metrics: Recall, Precision, F1 Score, ROC AUC, and Accuracy, displayed as a radar chart.

The results show that each model has specific strengths and limitations depending on the metric considered. For example, ARIMA and LSTM tend to achieve higher recall, while ETS and Prophet show higher precision or ROC AUC. As evidenced by Table 12, the Transformer model yields the highest PAM value (0.87), confirming its robustness across the combined assessment criteria. The Transformer model maintains higher values across most metrics, but does not always lead in every category. This diversity in performance highlights the importance of selecting a prediction model based on the specific requirements and priorities of the IoT application scenario. The radar chart in Fig. 6 provides a visual comparison, supporting a nuanced interpretation of each model’s behavior.

The AUPRC results in Table 12 further validate the architectural advantages of the evaluated frameworks. Models like Prophet and Transformer exhibit high AUPRC values (0.88 and 0.86, respectively), indicating a strong ability to maintain precision even at high recall levels in imbalanced conditions. Conversely, despite its high recall, ARIMA’s low AUPRC (0.18) confirms its struggle with false positives, demonstrating why accuracy and ROC AUC alone can be misleading in IoT anomaly detection scenarios.

### Comparative experimental results with existing simulators

For real-world data acquisition, the system interfaces with physical sensors, specifically utilizing the DHT11 module for temperature and humidity measurements. This module operates within a standard voltage range and provides data with defined accuracy for both humidity and temperature. Its integration with microcontrollers like the Arduino Uno is straightforward, typically involving a single-wire protocol for data transmission (Table 13).

**Table 13:** Comparison of SHiELD with existing IoT simulators and related simulation studies.

Simulator (Year)	Reported Results	Test Hardware	RT	Pred.	Perf. Mon.
ATEMU (2004)	12 real s per 1 sim s (120 nodes); linear memory use up to $\sim$50 MB	Desktop PC (unspecified)	No	No	No
SENSE (2005)	2$\times$ faster than NS-2 in routing simulations; memory $\sim$30 MB for 100 nodes	Desktop PC (unspecified)	No	No	No
MobIoTSim (2016)	Real-time MQTT; no memory/CPU reported; 1 message/s per sensor	Android smartphone	Yes	No	No
IoTSecSim (2024)	100 nodes: 5m42s; 1000 nodes: 1h33m; peak RAM: 15.6 GB	Intel i7-8850H / HPC cluster	No	No	No
Kaala (2022)	10 devices: $\sim$5% CPU, $\sim$300 MB RAM; 100 devices: $\sim$48% CPU, $\sim$1.8 GB RAM	VM: 2 vCPU, 4 GB RAM (Ubuntu)	Yes	No	No
EdgeMining Sim (2021)	500 devices: $\sim$60% CPU with edge analytics; memory not reported	High-performance workstation	Yes	Yes	No
WasteMgmt Sim (2024)	Route optimization saved $\sim$20% travel time; no system metrics	Desktop PC (assumed)	No	No	No
CloudExpert (2022)	No performance data; evaluated user satisfaction in simulator choice	Standard PC (assumed)	No	No	No
IoT Research Survey (2018)	No experimental results reported	N/A	No	No	No
TBM Simulator (2024)	Wear analysis: 15% reduction in cutter life prediction error; sim. time not reported	Laboratory setup with sensors	No	Yes	No
PHENotype Simulator (2023)	Drug candidate identification: 85% accuracy in COVID-19 repurposing; processing time varies	High-performance computing cluster	No	Yes	No
Driving Simulator (2025)	Behavioral analysis with real-time data collection; message valence effects	Driving simulator workstation	Yes	No	Yes
SHiELD – Local (2025)	CPU: 10–60%; RAM: $\sim$1 GB; avg. latency: 850 ms	Raspberry Pi 2 (ARM Cortex-A7)	Yes	Yes (ARIMA+)	Yes
SHiELD – External (2025)	CPU: 40–90%; RAM: $\sim$1.2–1.5 GB; avg. latency: 350 ms	Intel i7-9700K, 16 GB RAM	Yes	Yes (ARIMA+)	Yes

The comparative data indicates that some simulators report execution time and resource usage under specific experimental conditions. ATEMU emphasizes accuracy at the instruction level but reports a runtime of 12 real seconds for each simulated second when modeling 120 nodes. SENSE achieved improvements in simulation efficiency compared to NS-2, requiring around 30 MB of memory for simulations with 100 nodes. MobIoTSim provides data in real-time through MQTT but does not include system-level performance data.

IoTSecSim evaluated scalability across 100 to 1000 nodes and recorded memory usage close to 16 GB for the largest scenario. Kaala reported consistent growth in resource usage as the number of simulated devices increased, with 100 devices reaching nearly 50% CPU utilization on a constrained virtual machine. EdgeMiningSim integrated data analytics at the edge, reaching approximately 60% CPU utilization during intensive tasks.

Recent simulation studies further illustrate the diversity of application domains and evaluation strategies. The TBM Simulator^17^ focused on industrial equipment, reporting a 15% reduction in cutter life prediction error through the integration of experimental and numerical sensor data. The PHENotype Simulator^18^ demonstrated the use of large-scale phenotype data generation and predictive modeling to achieve 85% accuracy in identifying drug candidates for COVID-19, leveraging high-performance computing resources. The Driving Simulator^36^ enabled real-time behavioral analysis, capturing the effects of message valence and phone conversations on driver performance, with system metrics collected during controlled experiments.

The SHiELD tests conducted on a Raspberry Pi 2 demonstrated stable performance under variable loads, with CPU usage ranging between 10% and 60% and latency below 1 second. On desktop hardware, SHiELD operated with 40% to 90% CPU usage and an average latency of 350 milliseconds. Unlike the other simulators, SHiELD includes integrated ARIMA-based predictive modeling and allows monitoring of CPU, memory, and latency metrics during execution. Additionally, SHiELD uniquely generates a comprehensive reliability report, consolidating all relevant data quality, trustworthiness, and system performance metrics, an aspect not addressed by the other platforms or studies.

Among the evaluated platforms, only EdgeMiningSim also integrates data analysis in the simulation loop. However, it does not offer model-based forecasting nor embedded performance tracking. While Kaala and MobIoTSim provide real-time data transmission, they do not include forecasting or system introspection. The SHiELD framework, tested in both constrained and high-performance environments, was designed to support end-to-end processing with measurable system behavior during execution. The integration of forecasting enables the system to anticipate sensor data patterns based on historical values, which is beneficial in simulations involving failure scenarios, delay-sensitive decisions, or planning mechanisms that require advance estimation of future states. This capability, combined with the reliability reporting feature, contributes to a more realistic and proactive simulation environment, allowing researchers to evaluate not only how the system reacts to events, but also how it can prepare for them based on trends present in the data and the quantified trustworthiness of the simulation outputs.

### Report results

**Fig. 7:**
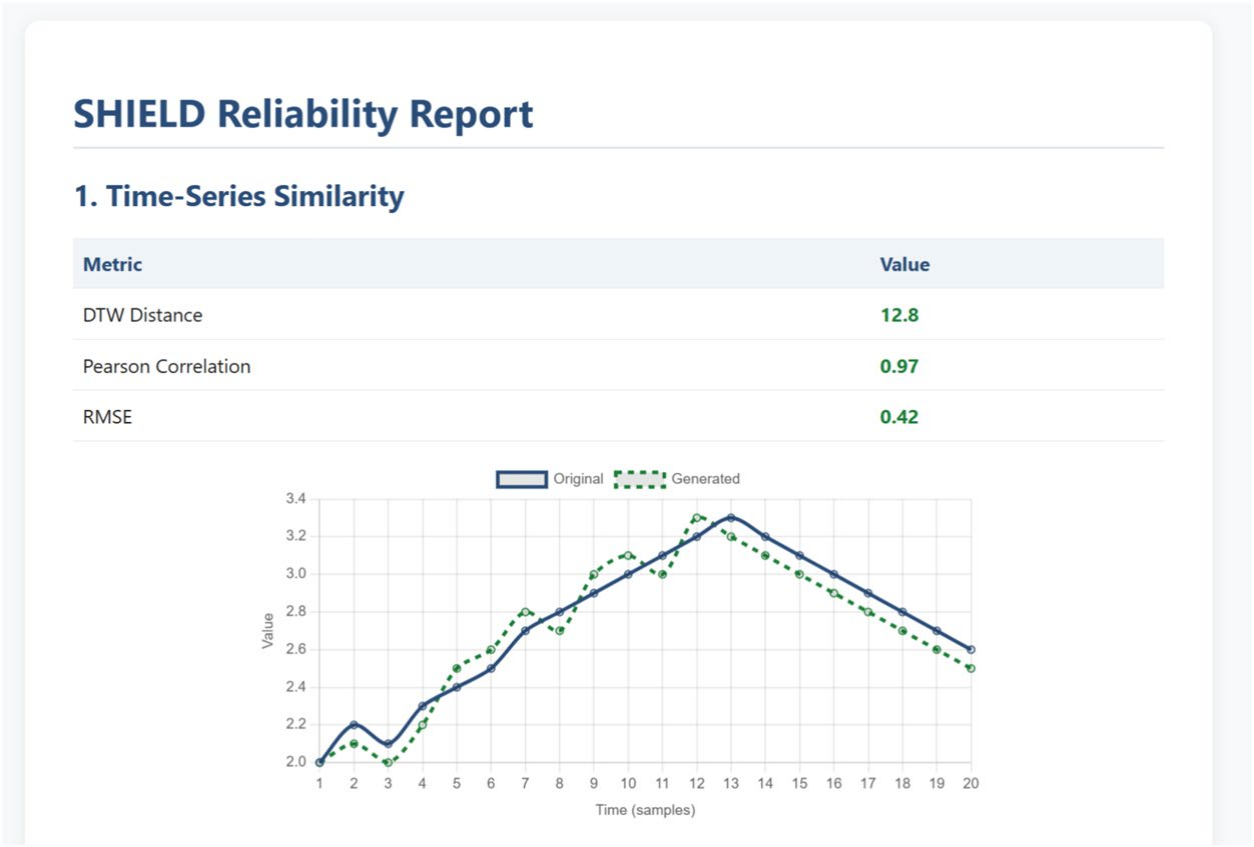
Similarity between the original and simulated.

**Fig. 8:**
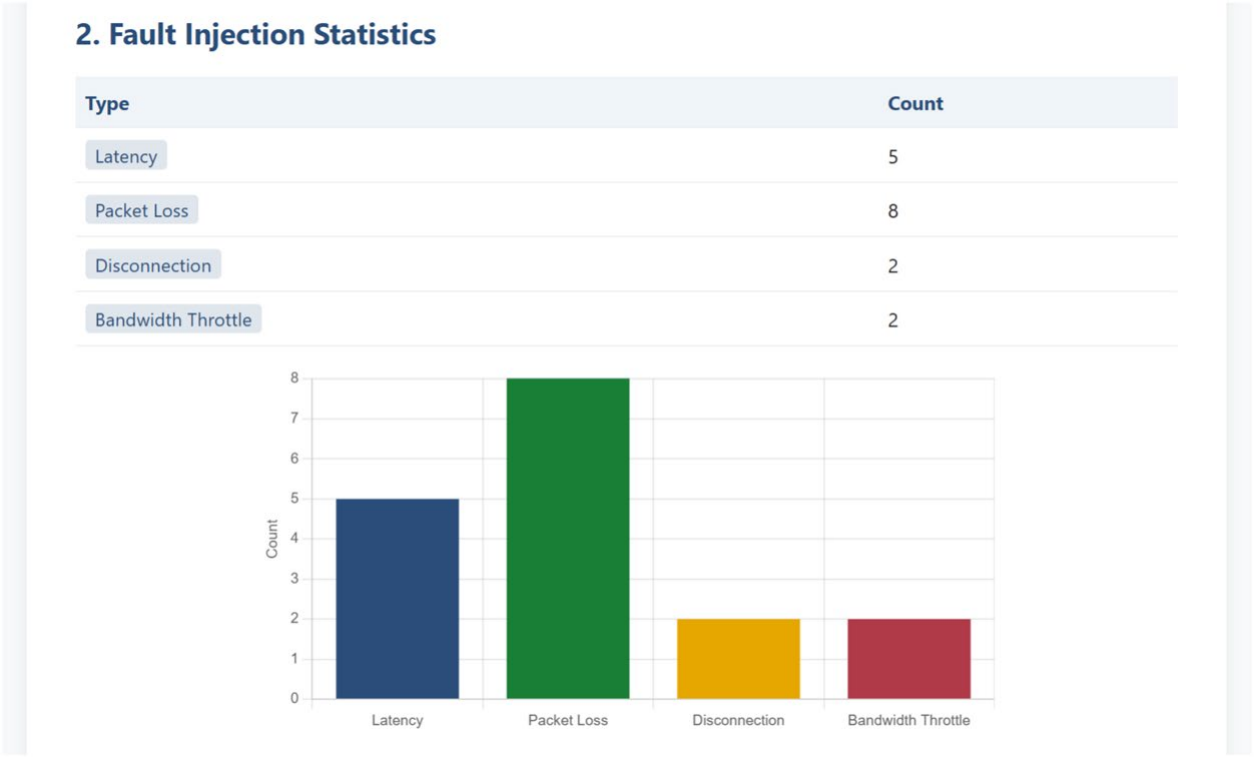
Fault injection statistics, showing the quantity and type of faults applied during the simulation.

**Fig. 9:**
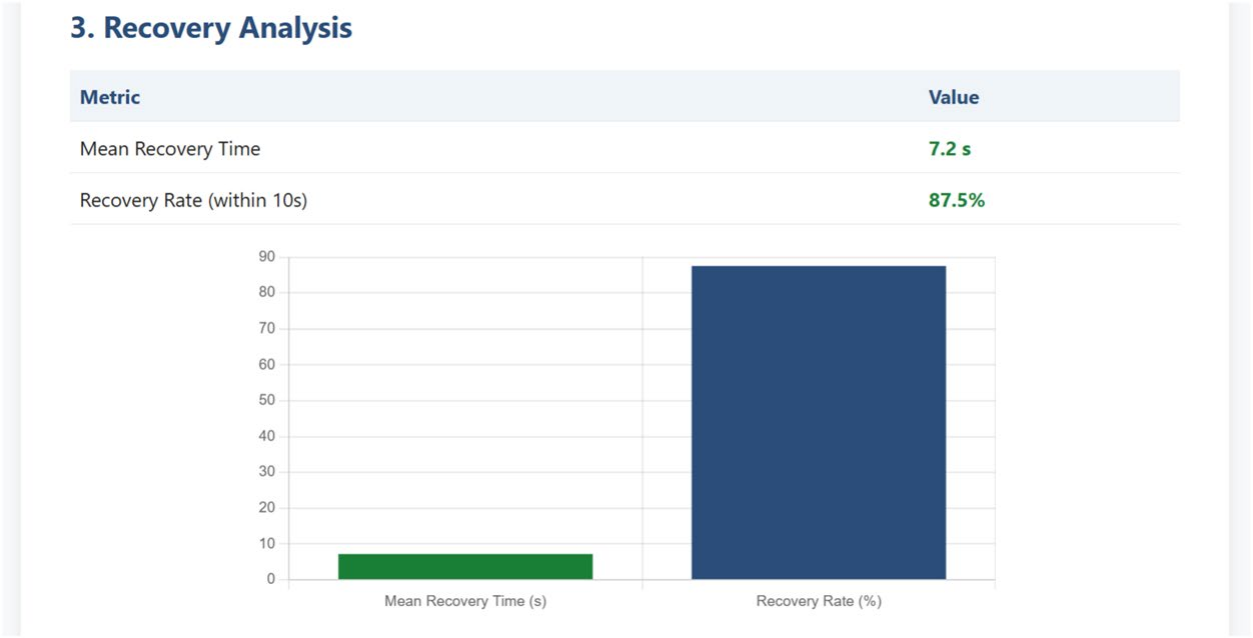
Recovery analysis, including mean recovery time and recovery rate after faults.

**Fig. 10:**
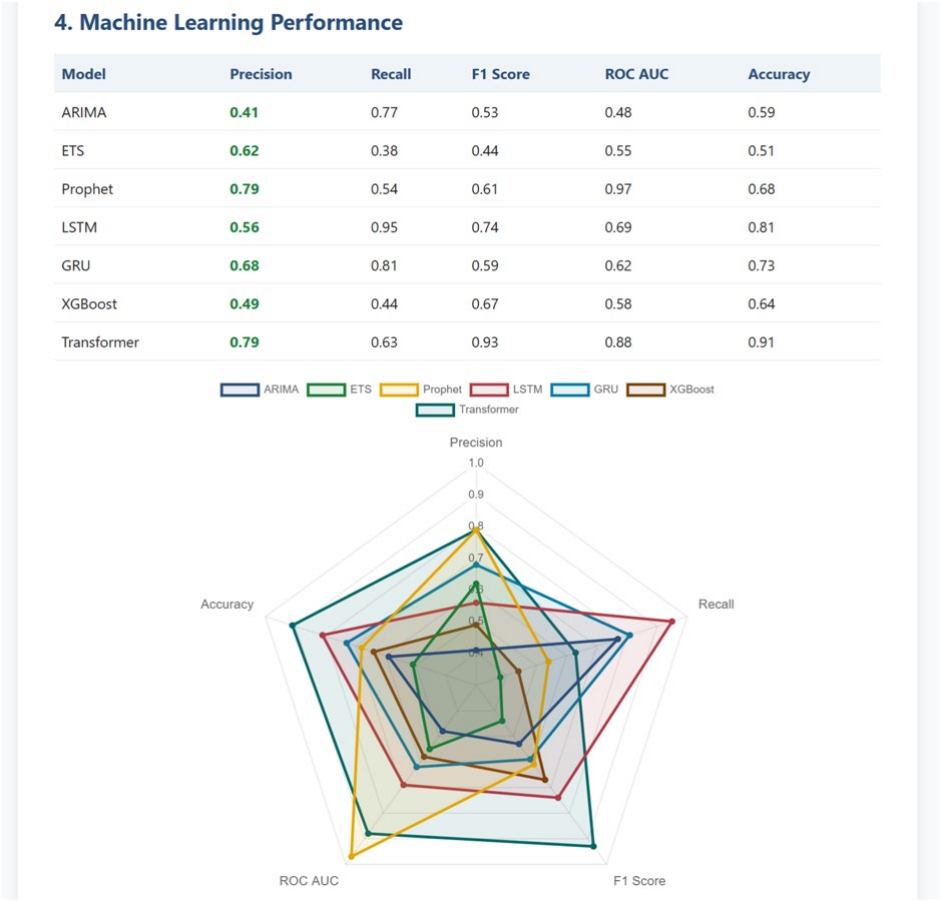
Performance of the evaluated ML models.

**Fig. 11:**
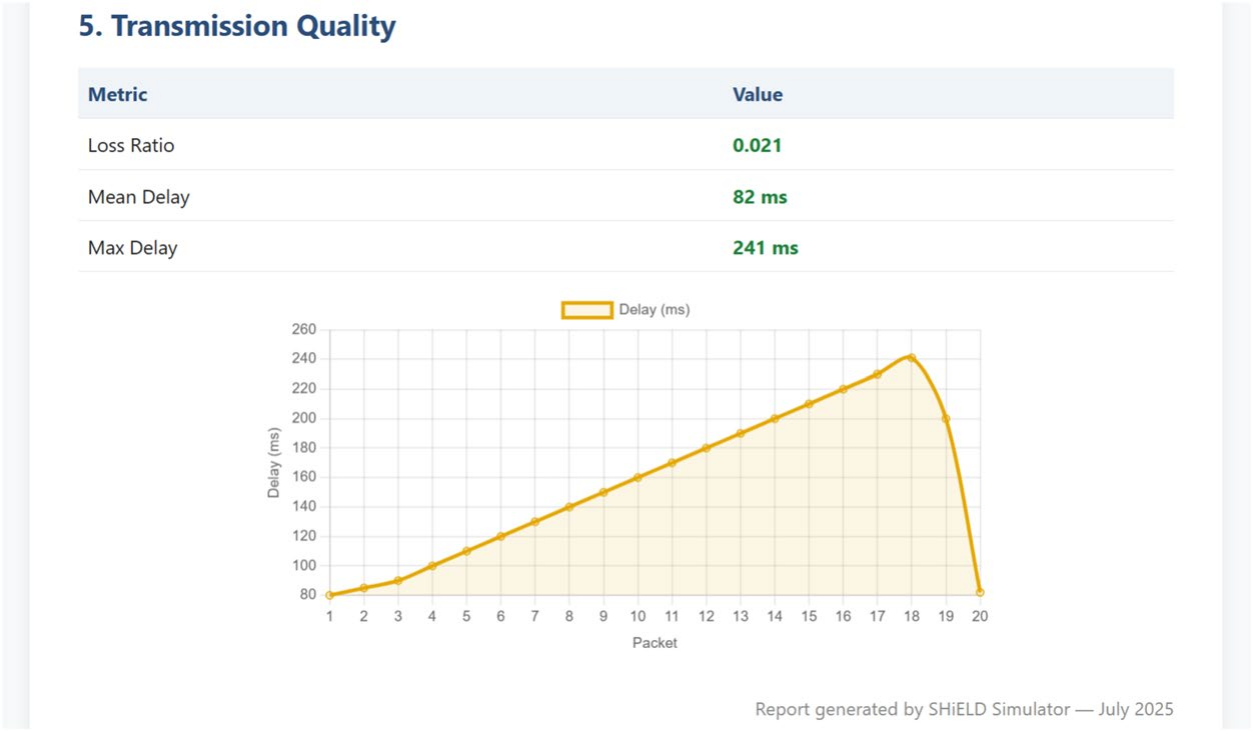
Transmission quality report.

This section presents the main outcomes of the reliability report generated by SHiELD, using the metrics discussed in the methodology. Figures 7, 8, 9, 10, and 11 illustrate, respectively, the time-series similarity, fault injection statistics, recovery analysis, machine learning model performance, and transmission quality. Figure 7 compares the original and simulated time series using metrics like DTW, Pearson correlation, and RMSE. The results show that the simulation captures key patterns from the real signal, with some differences in certain segments. These metrics help validate the simulation’s fidelity and indicate where parameter adjustments may be needed.

Figure 8 shows the frequency and types of faults injected during the simulation, such as latency, packet loss, disconnection, and bandwidth throttling. The chart highlights which faults were most common and their intensity, helping to assess the coverage of the test scenario. This information supports the analysis of each fault’s impact on system performance and resilience. Figure 9 details the system’s behavior after faults, presenting metrics such as mean recovery time and recovery rate within a predefined time window. The results show that the system resumes expected operation within an acceptable time in most cases, although variations occur depending on the type and severity of the fault. This analysis identifies possible bottlenecks in the recovery process and guides improvements in resilience mechanisms.

Figure 10 presents the performance of the different machine learning models used in the simulation, evaluated by metrics such as precision, recall, F1-score, ROC AUC, and accuracy. The chart enables comparison of model behavior with the simulated data, highlighting variations in performance among them. Some models achieve more consistent results in certain metrics, while others show greater sensitivity to specific aspects of the dataset. The diversity in model performance underscores the importance of benchmarking multiple approaches, as different models may excel under different data distributions or fault conditions.

Figure 11 presents the transmission quality metrics observed during the simulation, including packet loss ratio, mean delay, and maximum delay. The chart allows for the identification of intervals where network instability is more pronounced, often corresponding to periods when faults were injected. By examining these metrics, it is possible to evaluate how different types and frequencies of faults influence communication performance.

The data suggest that, while the system experiences some degradation during fault events, it generally operates within the expected parameters for the simulated scenario. This analysis helps to clarify the relationship between network conditions and overall system behavior, and provides a basis for assessing whether the current configuration meets the intended reliability and quality of service targets. The results offer a detailed view of the system’s behavior across various operational scenarios. By combining analyses of time-series similarity, fault injection statistics, recovery processes, machine learning performance, and transmission quality, the report supports a broad evaluation of the simulator.

The reliability reports generated by SHiELD provide practical information for developers and researchers. They present quantitative data on how the simulator responds to different conditions, which assists in validating model accuracy and understanding system limitations. By documenting the effects of injected faults and recovery actions, the reports help clarify which aspects of the system may benefit from further refinement. These reports contribute to ongoing development and validation efforts by offering clear, structured feedback on system performance. They also facilitate comparisons between simulation runs and support informed decisions regarding future improvements to the SHiELD platform.

Furthermore, to ensure a fair performance comparison, all models within the SHIELD framework are evaluated under strictly identical conditions, using the same input streams and fault-injection parameters; while external benchmarking against standardized IoT workloads is planned for future iterations to further verify architectural merit against other platforms. The comprehensive reliability report generated by SHIELD goes beyond raw data presentation by synthesizing key performance metrics into actionable insights. It integrates time-series similarity measures, fault injection statistics, recovery performance, and predictive model evaluations into a cohesive summary. Visual representations and concise statistics enable users to quickly identify system strengths and weaknesses, facilitating targeted improvements and reducing the need for extensive manual data interpretation.

Twenty independent simulation trials across varied fault injection profiles, including network latency, packet loss, and node outage scenarios, substantiate the robustness of the reliability assessments. The analysis aggregates core reliability indicators, Dynamic Time Warping (DTW) distance, Pearson correlation coefficient, system recovery time, and data loss ratio, using mean and standard deviation to demonstrate consistency under diverse conditions, rather than relying solely on illustrative examples (Table 14).

**Table 14:** Aggregated reliability metrics across simulation trials.

Metric	Mean	Standard Deviation
DTW Distance	13.1	0.4
Pearson Correlation	0.94	0.02
Recovery Time (s)	7.4	0.6
Loss Ratio	0.023	0.004

These results indicate that the SHiELD reliability reports deliver consistent, reproducible evaluations across multiple operational scenarios. The low variance in all metrics demonstrates the framework’s stability in characterizing system behavior under dynamic environmental perturbations.

## Conclusion

This work presents an integrated approach to simulating, processing, and analyzing sensor data in an IoT environment, addressing the need for tools capable of simulating the full lifecycle of sensor data. The developed simulator offers a platform for researchers and practitioners to generate, process, and predict sensor data in a controlled manner, while also supporting real-time monitoring and performance evaluation.

One of the key contributions of this study is the integration of heuristic-based data processing techniques, including aggregation, compression, and filtering, into the SHiELD system. These heuristics effectively reduce data volume while preserving critical information, thereby optimizing bandwidth usage and improving overall system efficiency. Specifically, aggregation reduced packet transmissions by up to 82.5%, significantly lowering communication overhead. Compression and filtering further decreased data size by approximately 9.4%, making the system more suitable for bandwidth-constrained IoT environments.

The integration of predictive models, including ARIMA and several state-of-the-art machine learning approaches such as LSTM, GRU, Transformer, Prophet, XGBoost, and ETS, further enhances SHiELD by enabling robust forecasting of sensor data trends. The comparative evaluation of these models, using the precision, recall, F1 score, ROC AUC, and precision metrics, revealed distinct performance profiles, supporting the selection of the most appropriate model for each application scenario. This multi-model approach increases the flexibility and applicability of the simulator across diverse IoT contexts.

The system includes mechanisms for simulating unpredictable network and system failures through tools such as ToxyProxy and custom fault injection layers, enabling the evaluation of resilience under adverse conditions. Reliability reports generated at the end of each run provide detailed insights into recovery times, recovery rates, fault impacts, time-series similarity, fault injection statistics, machine learning performance, and transmission quality, ensuring transparent and reproducible analysis. Validation on real hardware, including Arduino-based sensor data acquisition combined with processing on Raspberry Pi and server environments, confirmed the simulator’s adaptability to resource-constrained scenarios and its potential for real-world deployment.

The implementation of SHiELD also required addressing challenges inherent to real-time sensor data processing, including computational overhead, efficient message queuing, and asynchronous event handling. Integrating predictive models demanded balancing complexity with feasibility to maintain scalability in IoT contexts, while ensuring adaptability to diverse sensor configurations required a modular system design. These elements distinguish SHiELD from traditional simulators and highlight the need for structured, scalable approaches to real-time sensor simulation and prediction. Despite these promising results, several areas remain open for future research and system enhancement:

- *Adaptive Heuristics* automatic tuning of heuristics to match real-time data and variable loads, optimizing resource use.
- *Enhanced Security* add anomaly detection, secure communication, and stronger encryption to protect IoT data and privacy.
- *Comprehensive Dataset Testing* evaluate on diverse datasets and additional hardware (e.g., Arduino) to validate performance across scenarios.
- *Unpredictable Fault Simulation* broaden fault-injection coverage (ToxyProxy and custom faults) to test robustness under varied failures.

Addressing these improvements will facilitate the development of more robust, secure, and scalable IoT systems, capable of efficiently managing extensive sensor data and delivering reliable, timely analytics for critical decision-making.

## Data Availability

All the generated dat can be accessed in https://github.com/DarlanNoetzold/SensorSimulator.
